# CryoEM analysis of the essential native UDP-glucose pyrophosphorylase from *Aspergillus nidulans* reveals key conformations for activity regulation and function

**DOI:** 10.1128/mbio.00414-23

**Published:** 2023-07-06

**Authors:** Xu Han, Cecilia D'Angelo, Ainara Otamendi, Javier O. Cifuente, Elisa de Astigarraga, Borja Ochoa-Lizarralde, Martin Grininger, Francoise H. Routier, Marcelo E. Guerin, Jana Fuehring, Oier Etxebeste, Sean R. Connell

**Affiliations:** 1 Structural Biology of Cellular Machines Laboratory, Biocruces Bizkaia Health Research Institute, Cruces University Hospital, Barakaldo, Bizkaia, Spain; 2 Center for Cooperative Research in Biosciences (CIC bioGUNE), Basque Research and Technology Alliance (BRTA), Derio, Spain; 3 Structural Glycobiology Laboratory, Biocruces Bizkaia Health Research Institute, Cruces University Hospital, Barakaldo, Bizkaia, Spain; 4 Laboratory of Biology, Department of Applied Chemistry, Faculty of Chemistry, University of the Basque Country, UPV/EHU, San Sebastian, Spain; 5 Institute of Organic Chemistry and Chemical Biology, Buchmann Institute for Molecular Life Sciences, Goethe University Frankfurt, Frankfurt am Main, Germany; 6 Institute for Clinical Biochemistry, Hannover Medical School, Hannover, Germany; 7 Ikerbasque, Basque Foundation for Science, Bilbao, Spain; Karlsruhe Institute of Technology, Karlsruhe, Germany

**Keywords:** aspergillosis, cell wall biosynthesis, UDP-glucose pyrophosphorylases, enzyme mechanism, enzyme specificity, cryoEM

## Abstract

Invasive aspergillosis is one of the most serious clinical invasive fungal infections, resulting in a high case fatality rate among immunocompromised patients. The disease is caused by saprophytic molds in the genus *Aspergillus*, including *Aspergillus fumigatus*, the most significant pathogenic species. The fungal cell wall, an essential structure mainly composed of glucan, chitin, galactomannan, and galactosaminogalactan, represents an important target for the development of antifungal drugs. UDP (uridine diphosphate)-glucose pyrophosphorylase (UGP) is a central enzyme in the metabolism of carbohydrates that catalyzes the biosynthesis of UDP-glucose, a key precursor of fungal cell wall polysaccharides. Here, we demonstrate that the function of UGP is vital for *Aspergillus nidulans* (*An*UGP). To understand the molecular basis of *An*UGP function, we describe a cryoEM structure (global resolution of 3.5 Å for the locally refined subunit and 4 Å for the octameric complex) of a native *An*UGP. The structure reveals an octameric architecture with each subunit comprising an N-terminal α-helical domain, a central catalytic glycosyltransferase A-like (GT-A-like) domain, and a C-terminal (CT) left-handed β-helix oligomerization domain. *An*UGP displays unprecedented conformational variability between the CT oligomerization domain and the central GT-A-like catalytic domain. In combination with activity measurements and bioinformatics analysis, we unveil the molecular mechanism of substrate recognition and specificity for *An*UGP. Altogether, our study not only contributes to understanding the molecular mechanism of catalysis/regulation of an important class of enzymes but also provides the genetic, biochemical, and structural groundwork for the future exploitation of UGP as a potential antifungal target.

**IMPORTANCE:**

Fungi cause diverse diseases in humans, ranging from allergic syndromes to life-threatening invasive diseases, together affecting more than a billion people worldwide. Increasing drug resistance in *Aspergillus* species represents an emerging global health threat, making the design of antifungals with novel mechanisms of action a worldwide priority. The cryoEM structure of UDP (uridine diphosphate)-glucose pyrophosphorylase (UGP) from the filamentous fungus *Aspergillus nidulans* reveals an octameric architecture displaying unprecedented conformational variability between the C-terminal oligomerization domain and the central glycosyltransferase A-like catalytic domain in the individual protomers. While the active site and oligomerization interfaces are more highly conserved, these dynamic interfaces include motifs restricted to specific clades of filamentous fungi. Functional study of these motifs could lead to the definition of new targets for antifungals inhibiting UGP activity and, thus, the architecture of the cell wall of filamentous fungal pathogens.

## INTRODUCTION

The fungal cell wall is composed of a dynamic polysaccharide-based three-dimensional (3D) network that is continuously adapting to growth conditions and environmental stresses and is essential for cell survival (1). The central core consists of a branched β(1,3)-glucan with 3% to 4% interchain linked (via β(1,4) linkage) to chitin, a homopolysaccharide of β(1,4)-linked *N*-acetylglucosamine (GlcNAc) residues (2 - 6). This structural core can be differently decorated depending on the fungal species. In *Aspergillus* hyphae, the outer cell wall layer is composed of amorphous α(1,3)-glucan, galactosaminogalactan, galactomannan, and GPI (glycosylphosphatidylinositol)-anchored and surface proteins (6). The fungal cell wall provides an initial barrier to the hostile environment. It also harbors several hydrolytic and toxic activities necessary for a fungus to invade its ecological niche. In addition, its rigid structure, in combination with turgor pressure, is useful as a force for the penetration of insoluble substrates that it colonizes. The genus *Aspergillus*, which includes approximately 350 species, has a tremendous impact on public health both beneficially as the workhorse of industrial applications and negatively as plant and human pathogens (7, 8). *Aspergillus fumigatus* is the most common and opportunistic pathogen that causes 90% of invasive aspergillosis, with a 50%–95% mortality rate (9, 10). Infections with *A. fumigatus* are becoming increasingly resistant to azole antifungals, and recent studies suggest that resistant strains are transmitted from the environment, where they evolve as a result of exposure to agricultural azole fungicides (11). *Aspergillus flavus* and *Aspergillus parasiticus* can contaminate several common crops with aflatoxin, a highly toxic carcinogen with immunosuppressive properties (12). The consumption of contaminated crops can cause serious illness or death and is a common problem in developing countries. Additional species, including *Aspergillus niger*, *Aspergillus oryzae,* and *Aspergillus terreus*, are important in the food and pharmaceutical industry (13). *A. nidulans* is an opportunistic pathogen and a saprophyte and has served during the last 80 years as a reference system for aspergilli and close genera in the study of the molecular and genetic mechanisms controlling polar growth of hyphae, intracellular transport, stress response, development, secondary and primary metabolism, and cell wall assembly and processing (14). Fast and reproducible growth and developmental cycles were among the features that made *A. nidulans* a central reference filamentous fungus.

**Fig 1:**
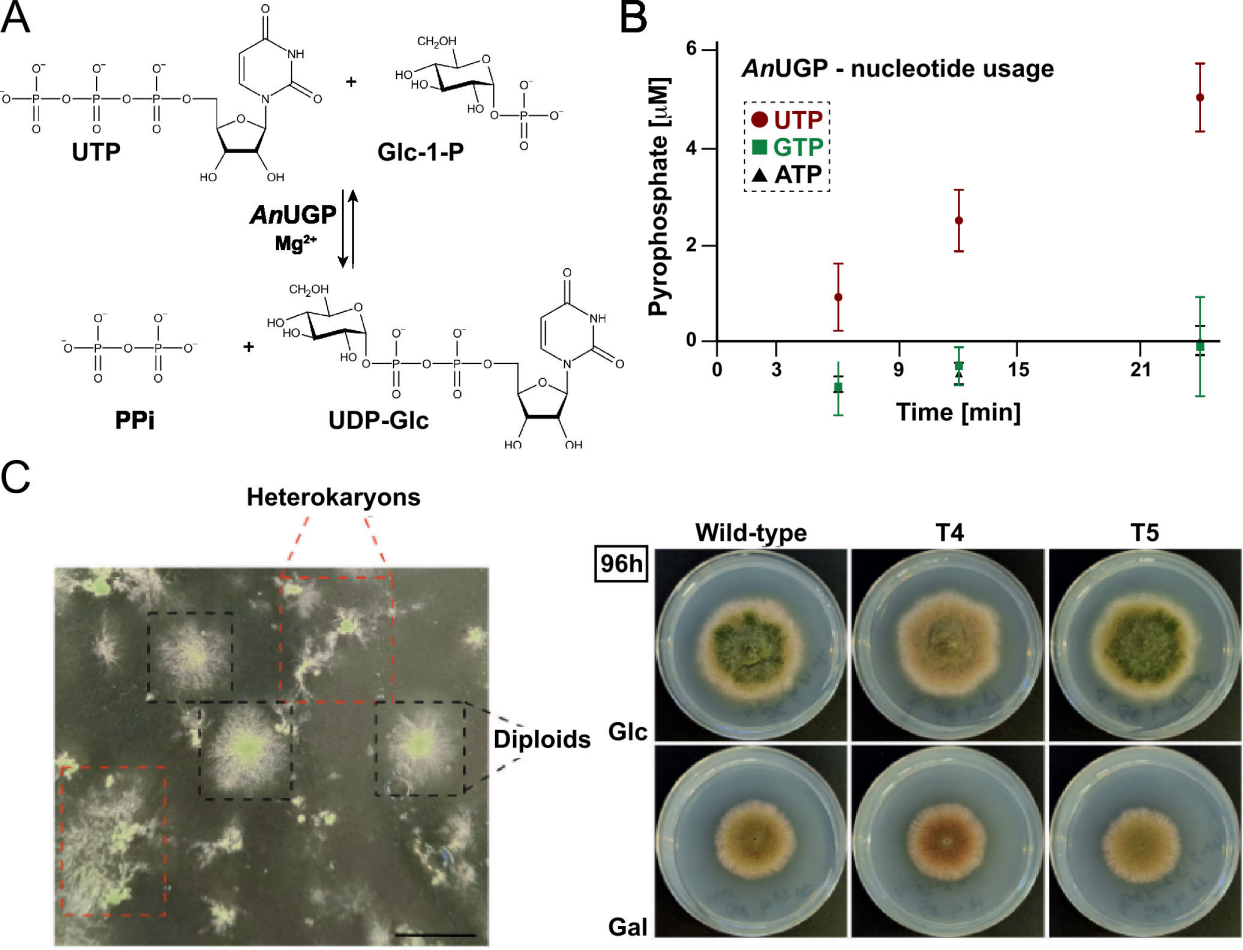
*An*UGP is an UGP and is essential in *A. nidulans*. (**A**) *An*UGP catalyzes the reaction between UTP and Glc-1-P in the presence of a divalent metal cation, Mg^2+^, to form UDP-Glc and pyrophosphate (PPi). The enzymatic reaction is reversible *in vitro*. However, the hydrolysis of inorganic PPi by inorganic pyrophosphatase results in an irreversible reaction *in vivo* in the direction of UDP-Glc biosynthesis. (**B**) *An*UGP is UTP-specific. Error bars represent the standard deviation of five experiments. (**C**) Left: Phenotype of the colonies grown on transformation plates (selective, lacking uridine and uracil, regeneration medium), after 72 h of culture at 37°C. Red squares highlight colonies with a characteristic heterokaryotic phenotype. Black squares indicate diploids. Scale bar = 1 cm. Right: Phenotype of two of the diploids (**T4 and T5**) on AMM supplemented with Glc (row 1) or Gal (row 2) as the main carbon source, after 96 h of culture at 37°C on 5.5 cm plates. *gal2* mutants were described to show a growth phenotype when Gal was used as the carbon source (22). Colony radii of the two diploids analyzed here are equal to that of the reference wild-type strain. The diploid nature of these two transformants is confirmed in Fig. S2.

UDP (uridine diphosphate)-glucose (UDP-Glc) plays a central role not only in the biosynthesis and assembly of the fungal cell wall, but also in carbohydrate metabolism associated with multiple cellular processes. UDP-Glc biosynthesis is initiated by the action of a hexokinase (D-hexose 6-phosphotransferase; EC, 2.7.1.1) which phosphorylates glucose (Glc) to generate glucose-6-phosphate (Glc-6-P). Then, Glc-6-P is converted to glucose-1-phosphate (Glc-1-P) by phosphoglucomutase (α-D-glucose 1,6-phosphomutase; EC 5.4.2.2). Finally, Glc-1-P is transformed into UDP-Glc by the action of the UDP-Glc pyrophosphorylase (UGP; UTP-glucose-1-phosphate uridylyltransferase; EC 2.7.7.9; Fig. 1A). The enzyme belongs to the ubiquitous family of NDP (nucleotide diphosphate)-sugar pyrophosphorylases (NSPs) (15 - 18), which are part of the nucleotidyltransferase superfamily (EC 2.7.7) characterized by a common core structure composed of a mixed β-sheet flanked by α-helices. NSPs provide activated donor sugars that serve as substrates for the synthesis of oligo- and polysaccharides as well as protein and lipid glycosylation. While most NSPs display specificity for both the NTP (nucleoside triphosphate) and sugar phosphate, some enzymes are capable of activating two or more sugars, such as UDP-GlcNAc/-GalNAc pyrophosphorylases (19) and UDP-sugar pyrophosphorylases (USPs) with broad substrate specificity identified in plants and some protozoan parasites [reviewed in reference (20)]. A second subclade of UGP, termed UGP-B, which was more closely related to USPs, was identified exclusively in plants, whereas the UGP-A subclade is found in all eukaryotes (21). NDP-activated sugars may be further converted by, e.g., epimerases, mutases, or oxidative pathways, giving rise to additional nucleotide-sugars. For example, the UGP reaction product UDP-Glc can be converted to UDP-galactose and UDP-glucuronic acid, making UGP one of three NSPs that are essential for the synthesis of the major fungal cell wall components.

In this work, we determine that the *galF* gene encoding the enzyme is an essential gene in *A. nidulans*. Furthermore, we isolate native UGP from *A. nidulans* (*An*UGP; previously known as GalF or Gal2; FungiDB ID AN9148), and report, to our knowledge, the first structure of a UGP based on cryoEM data. *An*UGP shows an octameric architecture with unprecedented conformational variability in the individual protomers. In combination with enzymatic activity measurements and computational analysis, we unveil the molecular basis of substrate recognition, specificity, and catalysis for *An*UGP, providing the groundwork for the future exploitation of UGP as a potential antifungal target.

## RESULTS

### *An*UGP mediates the biosynthesis of UDP-Glc in *A. nidulans*

Endogenous native *An*UGP was purified owing to its ability to bind a Ni-NTA affinity column without any added tag. The elution profile of the protein from a gel filtration column (Fig. S1) indicated it was an intact octameric complex. The activity of the isolated *An*UGP was measured in the direction of synthesis of the nucleotide-sugar and pyrophosphate (PPi), coupling the reaction with the hydrolysis of PPi and following the formation of Pi by malachite green (MG) assay (Fig. 1B). As expected, the enzyme was specific for UTP and unable to use ATP or GTP as donor substrate under the conditions used (Fig. 1B). The specific activity of the enriched enzyme, calculated per milligram of total protein, at 30°C, is 0.41 ± 0.06 µmol/mg·min. Altogether, the enzymatic activity measurements indicate that *An*UGP is a *bona fide* UGP.

### *An*UGP, encoded by the *galF* gene, is essential for growth in *A. nidulans*

Early genetic screens identified a series of *A. nidulans* mutants unable to grow when galactose (Gal) was used as the sole carbon source for the cultures (22). Among those mutants, *gal2* showed slow growth under these culture conditions and was thus classified as a Gal utilization mutant. The *A. nidulans* linkage map (https://www.fgsc.net/Aspergillus/gene_list/locigh.html) renamed *gal2* as *galF,* and the FungiDB database (23) annotated *galF* as encoding a putative UTP-glucose-1-phosphate uridylyltransferase; thus, we refer to the protein as *A. nidulans* UGP (*An*UGP) (24). UGP is required for Gal utilization. Specifically, the product of UGP, UDP-Glc, is used by galactose-1-phosphate (Gal-1-P) uridylyltransferase as a substrate for the conversion of Gal-1-P to Glc-1-P in the Leloir pathway (GALT; EC 2.7.7.12) (25).

To assess the essentiality of the gene *galF*, we generated a linear DNA construct in which the selection marker *pyrG^Afum^* (the *pyrG* gene of *A. fumigatus*) was fused to the promoter region (5´-UTR) and the 3´-UTR region of *galF* (see Materials and Methods). Protoplasts of strain TN02A3 were transformed with this construct. The TN02A3 strain bears a deletion of the gene *nkuA*, the homolog of the human *KU70* gene, which is essential for non-homologous end joining DNA in double-strand break repair and favors homologous recombination (26). Most of the colonies obtained on selective regeneration medium (RMM) displayed irregular and slower radial growth compared to wild type (Fig. 1C) and were unable to grow on selective AMM (standard *Aspergillus* minimal medium). These characteristics suggest that they were heterokaryons (27). Haploid and homokaryotic knock-out transformants would likely display an increased branching pattern as reported for RNAi knock-down mutants of the *ugp* homolog of the Agaricomycete fungus *Ganoderma lucidum* (28).

A few of the colonies obtained showed a stable phenotype and homogeneous radial growth, both on selective RMM and AMM culture media (Fig. 1C). In contrast to previously described *gal2* mutants (22), these isolated colonies did not show any growth defect on medium supplemented with Gal as the sole carbon source (Fig. 1C). Moreover, diagnostic PCR reactions indicated the presence of the *pyrG* cassette and the *galF* gene, strongly suggesting that those transformants were diploids (Fig. S2). Overall, these results indicate that none of the two types of transformants obtained were haploid and homokaryotic *galF* deletion mutants, and that *galF*, encoding *An*UGP, is essential for *A. nidulans* viability.

### Visualizing native *An*UGP by cryoEM

**Fig 2:**
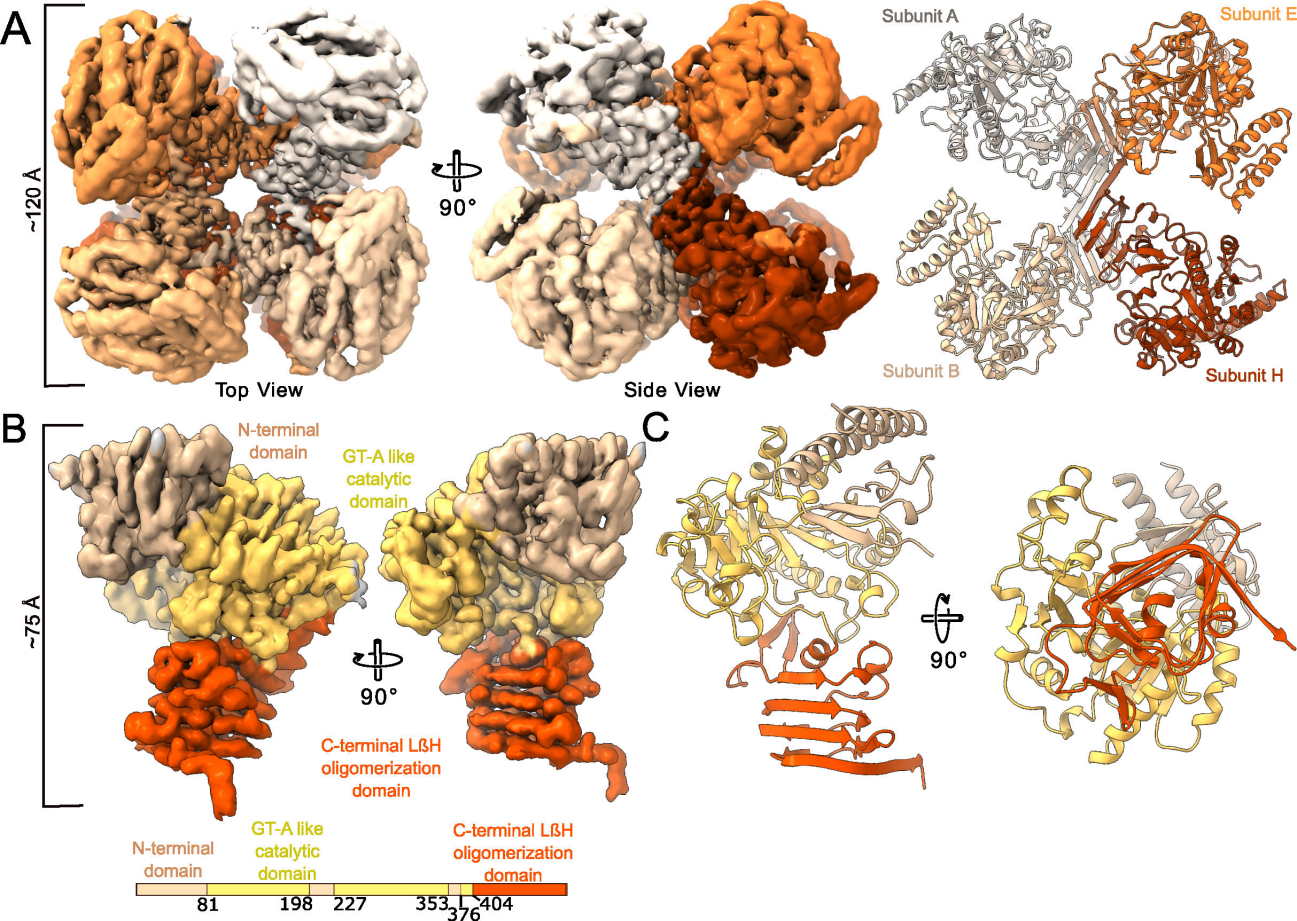
The native *An*UGP homoctamer visualized by cryoEM. (**A**) The cryoEM map (left, center) and model (right, ribbon representation) of the homoctameric *An*UGP refined with D4 symmetry are shown from the top and side views. The map has been colored to reflect the individual *An*UGP subunits. See Video S1 for superposition of map and model. (**B**) The cryoEM map and (**C**) model corresponding to a single *An*UGP subunit after local refinement are shown from various views. A schematic representation of the *An*UGP subunit domain architecture is shown, including the NT domain, the central GT-A-like domain, and the CT LβH oligomerization domain. The map and model have been colored according to domain structure in the schematic representation.

To understand the molecular mechanism of UDP-Glc biosynthesis at a structural and functional level, we determined the structure of *An*UGP (Uniprot code Q5I6D1) by cryoEM (Fig. 2; Tables S1 and S2; Fig. S3 ). Both the raw cryoEM images and the 2D class averages (Fig. S3A through C) show a particle with eight subunits arranged around a fourfold axis (top view) and a twofold axis (side view), suggesting it displays D4 symmetry. Each *An*UGP subunit (57.6 kDa protein; 514 amino acids) is composed of three domains: (i) the α-helical N-terminal (NT) domain (residues 1–81, 199–227, 354–376), (ii) the catalytic glycosyltransferase A-like (GT-A-like) domain [residues 82–198, 228–353, 377–404 (29 - 31)] containing the active site, and (iii) the C-terminal (CT) oligomerization domain (residues 405–514) containing a left-handed parallel β-helix (LβH; residues Leu461–His514; Fig. 2C). The eight subunits are readily seen in an unbiased *ab initio* cryoEM map and an unsymmetrized C1 map (Fig. S1D and E). In the C1 map, the density corresponding to one subunit appears weaker or more fragmented than the others, suggesting one subunit has higher conformational or occupational heterogeneity. To take advantage of the particle’s apparent symmetry and increase the resolution of the cryoEM map, we refined the data while applying D4 symmetry, resulting in a 3.98 Å structure (Fig. 2A; Video S1). Analysis of the map indicates that it displays a broad local resolution range, with the CT LβH oligomerization domain showing up to 3.2 Å resolution, while peripheral secondary structure elements in the NT and catalytic domains show resolution >5 Å (Fig. S3F). The low local resolution indicates that the peripheral regions display conformational and compositional variability that breaks the D4 symmetry. To account for this flexibility and improve the interpretability of the map, we used symmetry expansion to generate a new particle data set where the projections are replicated but rotated using the point group symmetry such that all eight subunits of the complex are aligned on a single subunit. Afterward, a restrained local refinement, focusing on this single subunit, yielded a 3.5 Å cryoEM map for the *An*UGP monomer with local resolution ranging from 3.0 to >6 Å (Fig. 2B and C; Fig. S3H and I). Accurate tracing of the backbone and positioning of the sidechains were possible in the best regions of the map where the local resolution was high, whereas only placement of secondary structure elements (e.g., helices) was possible where the resolution was low (Fig. S3F, H, J). Accordingly, we present a model for residues 32–82, 86–361, and 371–514 of *An*UGP (Video S1). Overall, cryoEM analysis of native *An*UGP in solution indicates it is a homoctamer with pseudo-D4 symmetry broken by flexibility between the CT oligomerization domains and the central catalytic domains.

A search for structural homologs using the DALI server (32) (see Materials and Methods) revealed NSPs with significant structural similarity to the *An*UGP subunit (Fig. S4), including (i) UGP from *Saccharomyces cerevisiae* [*Sc*UGP (33)]; (ii) UGP from *Saccharum* hybrid cultivar SP80-3280 [*Sh*UGP (34)]; (iii) UGP from *Arabidopsis thaliana* [*At*UGP (35)]; (iv) UGP from *Homo sapiens* [*Hs*UGP (36)]; (v) UGP from *Leishmania major* [*Lm*UGP (37)]; (vi) UGP from *Trypanosoma brucei* [*Tb*UGP (38)]; (vii) UDP-GlcNAc pyrophosphorylase (UGlcNAcP) from *Aspergillus fumigatus* [*Af*UGlcNAcP (39)]; (viii) UGlcNAcP from *Entamoeba histolytica* [*Eh*UGlcNAcP (40)]; (ix) UGlcNAcP from *Homo sapiens* [*Hs*UGlcNAcP (41)]; (x) UGlcNAcP from *Candida albicans* [*Ca*UGlcNAcP (42)]; (xi) UGlcNAcP from *Mus musculus* (*Mm*UGlcNAcP); (xii) bifunctional GlcN-1-P acetyltransferase/UGlcNAcP GlmU from *Mycobacterium tuberculosis* [*Mt*GlmU (43)]. This structural similarity to NSPs, and specifically to other eukaryotic UGPs, reinforces the observation that *An*UGP is a *bona fide* UGP (Fig. 1B).

### *An*UGP oligomerizes through the CT domain utilizing interfaces maintained in animal and fungal UGPs

UGPs exist in diverse oligomeric states, including monomers in protozoan parasites (38, 44) and plants (35, 45 - 47), dimeric and tetrameric bacterial UGPs (48, 49), and octameric species observed in the animal and fungal kingdoms (33, 36, 50 - 52). Whereas plant UGPs, which are active as monomers, were described to be transiently inactivated by dimerization (35, 45 - 47), octamers appear to be the active species of fungal and human UGPs (33, 52).

**Fig 3:**
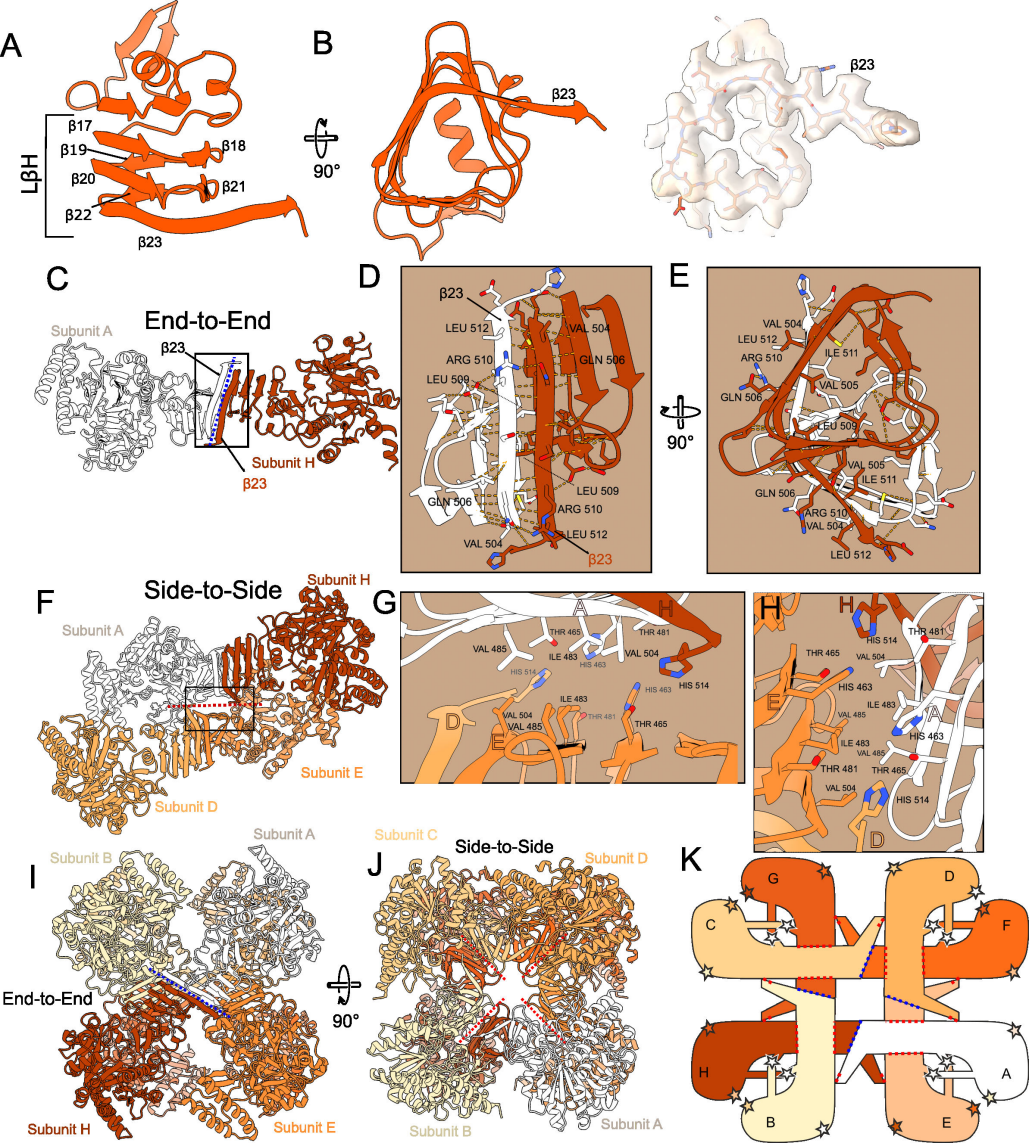
*An*UGP multimerization mechanism. (**A–B**) The CT LβH domain seen from two views with the latter view showing the cryo-EM map (local refinement) where sidechains are visible. (**C–E**) The end-to-end dimerization interface with two close-up views of the interface. Potential hydrogen bonds are shown as dotted lines. (**F–H**) The side-to-side dimerization interface, shown by a red dotted line, with two close-up views of the interface. (**I**) End-to-end dimerization interface (subunit A/H), shown by a blue dotted line, in the octameric complex (side view). (**J**) Side-to-side dimerization interfaces shown by red dotted lines in the octameric complex (top view). (**K**) Schematic representation of interaction interfaces in the octameric complex. Stars indicate potential dynamic interfaces, i.e., the latch-to-320-loop and the NT-to-β14/15-loop interfaces, described in Fig. 6.

**Fig 4:**
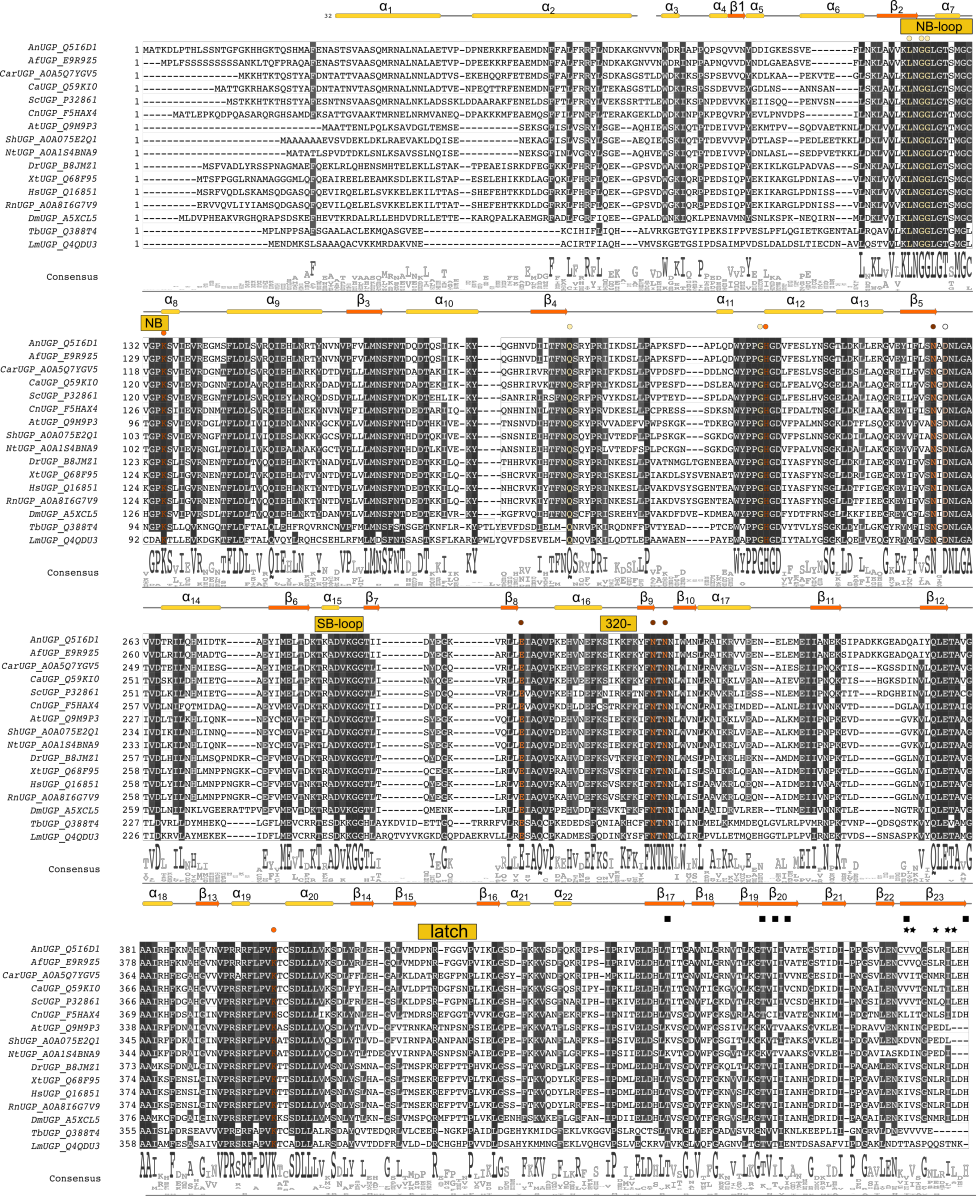
Sequence alignment of *An*UGP with selected UGPs. Protein sequence alignment (MUSCLE with default parameters) of selected UGPs. The sequences are ordered according to the identity, and residues that are at least 70% identical are highlighted in shades of black. Residues involved in UDP-Glc binding are marked with a colored circle and colored as in Fig. 5. Residues participating in the end-to-end interface are marked with a star, and those participating in the side-to-side interface are marked with a square. Secondary structures, as seen in the *An*UGP model, are shown above the alignment. UGPs for each organism are abbreviated as follows: *Aspergillus nidulans*, *An*UGP; *Aspergillus fumigatus*, *Af*UGP; *Candida auris*, *Car*UGP; *Candida albicans, Ca*UGP; *Saccharomyces cerevisiae, Sc*UGP; *Cryptococcus neoformans, Cn*UGP; *Arabidopsis thaliana, At*UGP; *Saccharum* hybrid cultivar, *Sh*UGP; *Nicotiana tabacum, Nt*UGP; *Danio rerio*, *Dr*UGP; *Xenopus tropicalis*, *Xt*UGP; *Homo sapiens*, *Hs*UGP; *Rattus norvegicus, Rn*UGP, *Drosophila melanogaster*, *Dm*UGP; *Trypanosoma brucei, Tb*UGP; *Leishmania major, Lm*UGP.

Octameric *An*UGP can be described as a tetramer of homodimers (Fig. 3) held together by two interfaces on the CT LβH (53), which is built of short β-strands (β17–β23) oriented parallel to each other and describing a triangular prism (Fig. 3A and B). The cryoEM map of the LβH region shows high local resolution (up to 3.2 Å; Fig. 3B; Fig. S3F, G, H), allowing the oligomerization interface to be described. Two *An*UGP subunits build the homodimer through “end-to-end” contacts between their C-terminal extended β-strands (Fig. 3A and B), forming an extended intermolecular β-sheet and a joint β-helix (Fig. 3C through E, I, K). This interface involves (i) antiparallel pairing of the final extended β23-strands, which creates an extensive potential hydrogen bond network between the backbones, and (ii) hydrophobic residues like Val504, Val505, Leu509, Ile511, and Leu512 (Fig. 3D and E). Next, the dimers engage in “side-to-side” contacts via one face of the LβH, which includes uncharged and hydrophobic residues like Thr465, Thr481, Ile483, Val485, and Val504, to form a tetramer of dimers (Fig. 3F through H, J, and K). Additionally, in the “side-to-side” interface (Fig. 3F through H), the rings of His463 from both partners lie “face-to-face” (subunits A and E), while the two CT His514 from each partner’s “end-to-end” pair (subunits H and D) approach the His463 stack end on, such that four subunits participate in the “side-to-side” interface. Overall, this oligomerization mode is preserved in other fungal and human UGPs (33, 36, 54) (Fig. S5A and B). All residues known to participate in both the “end-to-end” and “side-to-side” contacts are either strictly or functionally conserved in *An*UGP, likely supporting an equivalent function in *Sc*UGP (33) and *Hs*UGP (36, 54) (Fig. 4). Interestingly, extensive mutagenesis performed on the *Hs*UGP LβH domain (Table S3C) suggests that individual or simultaneous mutation of *An*UGP S508 and/or L509, as well as truncation of the CT eight amino acids, would result in dissociation of the octameric complex, which in the case of *Hs*UGP was associated with a dramatic drop in activity (52).

### *The An*UGP active site and enzymatic mechanism

**Fig 5:**
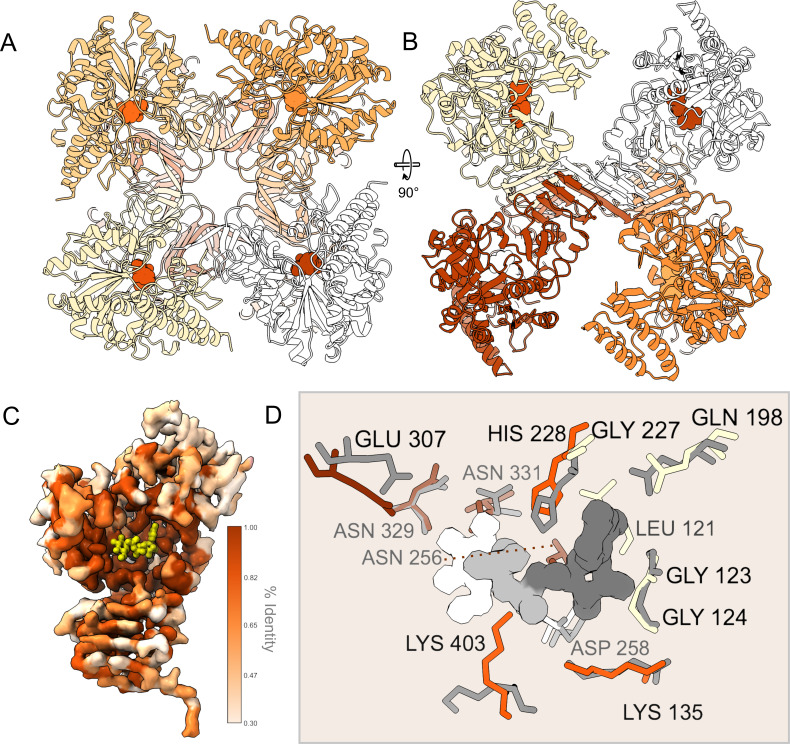
The *An*UGP active site is conserved with eukaryotic UGPs. (**A–B**) The inward-facing Glc-binding pockets are shown on the *An*UGP octamer from top and side views. The UDP-Glc is modeled by aligning PDB 4R7P. (**C**) Conservation (% identity) mapped to cryoEM map. (**D**) Comparison of functionally important residues in UDP-Glc pocket [*An*UGP and *Hs*UGP (PDBID 4R7P)]. *Hs*UGP residues are shown in gray. *An*UGP residues involved in binding uridine moiety are pale yellow (Leu121, Gly123, Gly124, Gln198, Gly227), sugar binding are brown (Asn256, Asn329, Asn331, Glu307), Mg^2+^ binding are white (Asp258), and phosphate biding are orange (Lys135, His228, Lys403). The UDP-Glc, from PDB 4R7P, is shown as a surface where the Glc is white, the phosphate groups are light gray, and the uridine moiety is dark gray. Owing to lower local resolution, the conformation of the *An*UGP sidechains cannot be determined unequivocally and should be considered possible conformations. The cryoEM map for Lys403, in particular, indicates that this sidechain is disordered in the apo *An*UGP structure.

UGPs catalyze the interconversion of UTP and Glc-1-P to UDP-Glc and PPi in a Mg^2+^-dependent reaction, following an ordered sequential Bi-Bi mechanism in which the uridine compound is the first to enter and the last to exit the active site (Fig. 1A; Fig. S6) (44, 54 - 57). To identify the *An*UGP active site and gain insight into the catalytic mechanism of *An*UGP, we compared the binding pocket of *An*UGP with *Hs*UGP (Fig. 5). The UDP-Glc binding pocket is in the GT-A-like domain, which consists of one Rossmann fold (58). The UDP-Glc binding site faces toward the inner cavity of the complex with four pockets each in both the upper and lower rings (Fig. 5A and B). As seen in Fig. 5C, mapping the conservation of *An*UGP on the cryoEM map shows that the region around the UDP-Glc binding pocket is highly conserved. Based on protein sequence alignments (Fig. 4) and previous structural/functional studies using X-ray crystallography (33, 35, 36, 54, 59, 60), we propose that in *An*UGP, (i) the Glc binding pocket is formed by Asn256, Asn329, and Asn331, (ii) the three conserved basic residues, Lys135, His228, and Lys403, coordinate the phosphate groups, (iii) Asp258 is involved in the coordination of a Mg^2+^ (57, 61), and (iv) the binding site for the uridine moiety is formed by Leu121, Gly123, and Gly124 in the nucleotide-binding (NB) loop, as well as Gln198 and Gly227. In addition to being strictly conserved in *An*UGP (Fig. 4), these residues are positioned similarly to those in *Hs*UGP (Fig. 5D), supporting a common substrate/product binding and catalysis for eukaryotic UGPs. As shown above, *An*UGP displays a strong preference for UTP (Fig. 1B) and, accordingly, in the structure, it is evident that the presence of small residues, Gly123 and Gly124, in the NB loop is vital for reducing steric clashes with the uridine moiety (Fig. 5D). Previous mutation of the residues corresponding to Gly123 and Gly124 in *Hs*UGP led to the loss of UGP activity (54) in agreement with structural studies on *Lm*UGP suggesting these positions are restricted to glycine for steric reasons (59).

### Conformational flexibility in the *An*UGP subunits

The *An*UGP cryoEM maps do not show proper D4 symmetry, but instead, the decreased local resolution in the NT and catalytic domains (Fig. S3F, H, J) suggests that these regions exhibit local flexibility that breaks the symmetry. Similarly, if the cryoEM-derived model of *An*UGP is compared with the X-ray models of closely related UGPs, it is evident that the relative arrangement of the NT/central and CT domain can vary (Fig. S5C). One power of cryoEM lies in the fact that before vitrifying the sample, the protein is maintained in a solution mimicking its native state where it can sample its accessible conformational space (62). Therefore, this distribution of molecular conformations remains after freezing, and the resulting reconstruction represents an amalgamation of preferred low-energy states (62).

**Fig 6:**
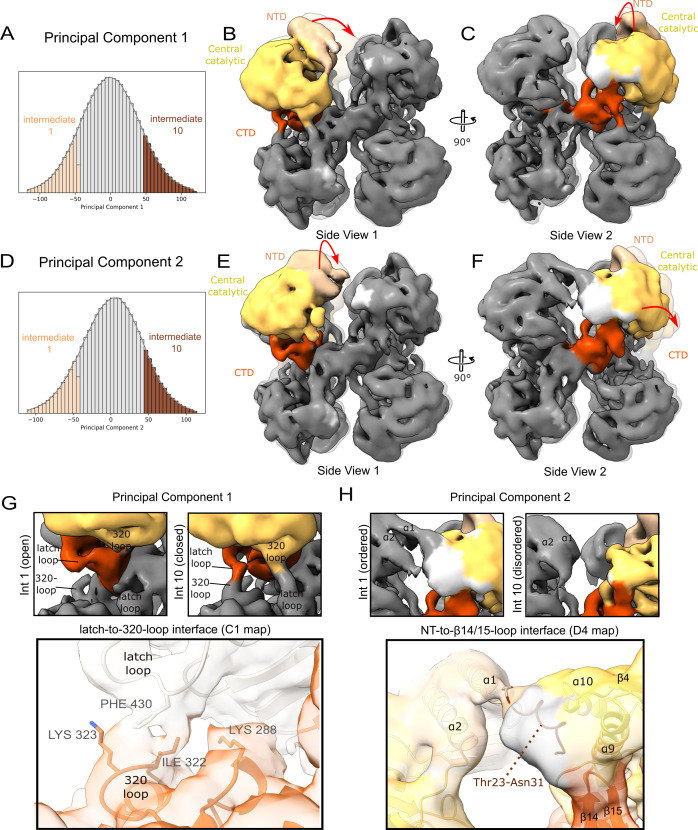
Conformational flexibility in *An*UGP. (**A**) The distribution of particles along PC 1. Particles selected for reconstruction of intermediates (int) 1 and 10 are colored distinctly. (**B–C**) Reconstruction of intermediates 1 (solid) and 10 (transparent) along PC 1 is superimposed and shown from two orthogonal views. The subunit that is the target of the 3DVA is colored according to the *An*UGP domain as defined in Fig. 2. The maps are low-pass filtered to 8 Å. (**D–F**) The same as seen in panels A–C but for PC 2. (**G**) Close-up view of the latch loop (Val424–Val433)/320-loop (Lys320–Lys326) interaction that forms (closed) and breaks (open) in intermediates 1 and 10, respectively. This “closed” interaction is also seen in some monomer pairs in the sharpened C1 map and potentially could include the interlock residue Lys288 which is part of the sugar-binding loop (54). The C1 map was selected for illustration as it is the least processed and most clearly shows interaction. Sidechains are shown for illustration purposes only as the local resolution does not allow for unequivocal positioning. (**H**) Close-up view of the interaction between *An*UGP NT residues and residues forming the loop between α10 and β4, the side of helix α9, and the short loop between β14 and β15.

To explore the variability in the *An*UGP monomer captured during freezing, we applied 3D variability analysis (3DVA) (63) to the symmetry-expanded data set before local alignment while using a mask to focus the analysis on the single monomer. 3DVA describes the variability in the data by solving for a continuous family of 3D structures and assigns each particle to a position within the principal component (PC) space. In this analysis, we observed that the first two PCs described clearly interpretable conformational changes in *An*UGP. Importantly, we did not observe discrete clusters in the PC space, indicating that the heterogeneity in *An*UGP was continuous rather than discrete, namely, in the *apo An*UGP structure, there are no dominant conformations present. To visualize the continuous conformational change, a “rolling window” is used to select particles along each PC axis. These particle subsets, termed intermediates, are reconstructed so movement along the PC axis can be described as a series of structures (Fig. 6A through F; Video S2). In Fig. 6, structures from the extremes of each PC axis, intermediate 1 (solid) and intermediate 10 (transparent), are compared. Comparing the two intermediates of PC 1 (Fig. 6B and C) shows that the NT/central domains of the monomer swing in a left-to-right motion in view 1 (Fig. 6B). The continuous movement along PC 1 axis can be seen in Video S2, where all 10 intermediates are shown. PC 2 shows an orthogonal swinging where in view 2 (Fig. 6F), the NT/central domains move left to right; this corresponds to an in-and-out movement in view 1 (Fig. 6E).

The orthogonal swinging motions alter the interface with the neighboring subunits as judged by the presence of continuous density bridges between two elements in the intermediate cryoEM maps (low-passed filtered to a common resolution of 8 Å). For example, a bridge is observed between regions of the intermediate 1 cryoEM map corresponding to loop Asp426-Pro434, which is termed latch loop owing to its overlap with the latch loop in *Hs*UGP (36), of one monomer and loop Lys320–Lys326 [320-loop; encompassing the 309 loop in *Hs*UGP (36)] of the neighboring monomer (Fig. 6G). Importantly, the swinging motion of the monomer breaks the bridge in the map of intermediate 10 (Fig. 6G; Video S2), indicating that the interface, termed latch-to-320-loop, is dynamic. The interaction is also seen in the less processed C1 (Fig. 6G) and D4 (not shown) cryoEM maps, where the superposition of the model clearly indicates residues of the latch- and 320-loop could be involved. A similar interaction was observed in the static crystal structure of *Hs*UGP (36), and the authors correlate it with differences in enzymatic activity between octameric UGPs, a so-called latch effect.

In intermediate maps (Fig. 6H), a continuous density bridge also forms through the partial ordering of some unmodeled NT residues of *An*UGP (1 - 31), extending the density at the N-terminus of α1 to reach the neighboring subunit at a junction between the catalytic and CT oligomerization domain. This potential interface on the neighboring subunit would be formed by the non-conserved loop between α10 and β4, the side of helix α9, and the short loop between β14 and β15 (Fig. 6H). This contact, termed NT-to-β14/15-loop, is also readily seen in the C1 (not shown) and D4 cryoEM maps (Fig. 6H). Although local resolution of the cryoEM does not allow reliable modeling of these NT residues, fewer than 10 residues would be required to span this bridge if intrinsically unstructured (Fig. 6H). AlphaFold (64, 65) indicates that in *An*UGP, residues 1–23 are unstructured, while 23–32 may have some α-helical character. The alignment in Fig. 4 shows that most UGPs do have similar extensions at the NT but the sequence is not conserved. Moreover, this NT interaction has not been observed in previous crystal structures of UGPs where the first 4 to 41 NT residues can be disordered (33 - 54 - 59 - 54 - 59), potentially due to crystal contacts or NT affinity tags. In summary, our analysis indicates that individual monomers experience conformational freedom inside the octameric assembly, and this variability can certainly influence interactions with neighboring monomers creating a dynamic set of intermolecular interfaces in addition to the more static “end-to-end” and “side-to-side” interfaces that are responsible for holding the monomers together in the octameric assembly (Fig. 3K).

## DISCUSSION

Fungi cause diverse diseases in humans, ranging from allergic syndromes to superficial/life-threatening invasive fungal diseases, which together affect more than a billion people worldwide (66). Very recently, the World Health Organization (WHO) provided the fungal priority pathogens list, which represents the first global effort to systematically prioritize fungal pathogens, considering their research and development needs and perceived public health importance (WHO report 2022; https://www.who.int/publications/i/item/9789240060241). The critical group includes *Cryptococcus neoformans*, *Candida auris*, *Candida albicans*, and *A. fumigatus*. To date, only four classes of systemic antifungal medicines—azoles, echinocandins, pyrimidines, and polyenes—are used in clinical practice, and only a few others are under development. The increased incidence of drug-resistant strains of *Aspergillus* species, especially for azole-resistant *A. fumigatus* infections, represents an emerging global health threat. Under such circumstances, the design of novel antimicrobial agents with mechanisms of action different from those of existing drugs has become a worldwide priority. In that context, enzymes participating in fungal cell wall biosynthesis and remodeling are attractive drug targets due to their essential biological roles. In this study, we demonstrate that in *A. nidulans, galF* (FungiDB ID AN9148) is an essential gene encoding a UTP-specific UGP, referred to as *An*UGP, a central enzyme in the metabolism of carbohydrates and assembly of the cell wall. Similarly, UGP was shown to be essential in *S. cerevisiae* (67) and the two filamentous fungal species *Ganoderma lucidum* (28) and *Grifola frondosa* (68); in all studies, downregulation of UGP activity was associated with cell wall defects and abnormal growth phenotypes.

**Fig 7:**
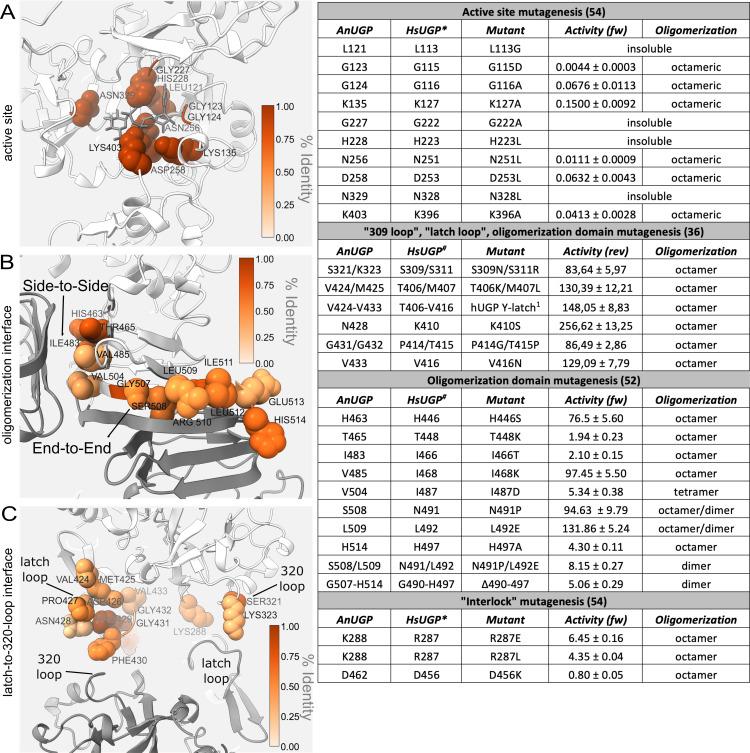
Functionally and structurally relevant sites in octameric UGPs. The functional and structural significance of key regions in the UGPs has been established by previous mutagenesis and enzymatic studies in *Hs*UGP (36, 52, 54). Results from these studies have been summarized in the table (see Table S3 for more extensive data) and the corresponding residues shown on the *An*UGP structure, highlighting (**A**) the active site, (**B**) the oligomerization interface, and (**C**) the dynamic latch-to-320-loop interface. The residues mentioned in the table are drawn with a sphere representation and colored according to percent identity (based on alignment in Fig. 4). The high conservation of these regions, in particular the active site and oligomerization interface, suggests that their significance is maintained in fungi (Fig. S7 ). In the table, numbering corresponds to the long *Hs*UGP isoform (*Hs*UGP*; 508 AA) or the short isoform (*Hs*UGP^#^; 497 AA). All activities are given as percentage of wild type, and fw corresponds to the forward reaction (UDP-Glc formation) and rev to the reverse reaction. The superscript 1 indicates that the *Hs*UGP latch loop was exchanged for that of *Sc*UGP.

We describe a cryoEM structure of a UGP and show that the fold of the *An*UGP monomeric unit is similar to that seen in crystal structures of eukaryotic UGPs (Fig. S5). Importantly, we harness the strength of cryoEM and analyze the conformational variability of the enzyme. Namely, we employ 3DVA to describe the major modes underlying the movement of an individual subunit in the complex. This movement alters the interaction with the neighboring subunit, and we observe changes in the interface between the latch loop, the 320-loop, and the SB (sugar-binding)-loop (Thr287–Thr295), as well as an interface between the NT residues and the neighboring subunit near the junction of the catalytic and CT domains. The identified active site, oligomerization, and dynamic interfaces (latch loop, the 320-loop, and the SB-loop) are critically important for the structure and enzymatic activity of UGPs (Fig. 7). Interestingly, the active site and oligomerization interfaces are more highly conserved, while the dynamic interfaces show more sequence variation (Fig. S7). The study of the functional significance and sequence divergence in the dynamic interface could provide the opportunity to design organism-specific regulators/inhibitors of UGPs.

### The catalytic mechanism of *An*UGP

Based on the sequence and structural conservation of *An*UGP with other eukaryotic UGPs (Fig. 4 and 5), we propose the UGP activity of *An*UGP follows the mechanism outlined in Fig. S6. The catalytic mechanism of *Lm*UGP was studied in detail, revealing that the basic residues, corresponding to *An*UGP Lys135, His228, and Lys403 (Fig. 5D), play crucial roles in catalysis (60). In mutagenesis studies, the *Lm*UGP and *Hs*UGP residues corresponding to Lys135 and Lys403 were essential for activity, whereas mutation of the homologous residue of His228 rendered *Lm*UGP almost inactive and *Hs*UGP insoluble (Table S3A) (54, 59). Assuming that eukaryotic UGPs follow a similar mechanism, *An*UGP Lys135 would form salt bridges with the β- and γ-phosphates of the substrate UTP, provide electrostatic stabilization of the transition state, and coordinate the byproduct PPi after the reaction (60). His228 likely coordinates the phosphate group of Glc-1-P before and during the reaction and the β-phosphate group of the product UDP-Glc (60). In *Hs*UGP, the sidechain of the corresponding histidine is rotated by 90° between the unbound and the UDP-Glc bound states, which is necessary to allow the formation of an H-bond with β-phosphate (54). Lys403 is expected to coordinate both the Glc-1-P phosphate and UTP α-phosphate, provide electrostatic stabilization of the transition state, and form hydrogen bonds to the α- and β-phosphates of the product UDP-Glc (60). The *An*UGP active site residue Asp258 is strictly conserved not only in eukaryotic but also in bacterial UGPs, where it was shown to be involved in coordination of a Mg^2+^ ion located between the UDP-Glc phosphate groups (57, 61). In *Lm*UGP, the Mg^2+^ ion was shown to facilitate the S_N_2 nucleophilic attack of Glc-1-P on the α-phosphate group of UTP and cleavage of the PPi leaving group by increasing the polarization and electrostatic attraction of the reacting atoms as well as favorably affecting the local geometry of the reaction center (60). Mutation of the corresponding residue in *Hs*UGP yielded an inactive enzyme, although the crystal structure of the *Hs*UGP-UDP-Glc complex does not contain a Mg^2+^ ion, and the aspartate appears to coordinate the ribose moiety of the product (54). Accordingly, *An*UGP Asp258 is likely to be enzymatically important and play a role in Mg^2+^ coordination and/or ribose binding.

In contrast to these residues, whose role in catalysis is strongly supported by mutation and structural studies in diverse systems including related octameric UGPs, other residues like *An*UGP Lys292 and Glu307 have more uncertain roles since their counterparts have been primarily studied in monomeric UGPs. *An*UGP Lys292, which is located within the SB-loop (Thr287–Thr295; Fig. 4), may play a role in coordinating the UDP-Glc β-phosphate, like its homologous residue in *At*UGP (35). However, this is inconclusive as in the *An*UGP cryoEM map, the sidechain of Lys292 is not well resolved, similar to the situation in *apo Hs*UGP and *Sc*UGP structures (33, 36), mostly likely due to the absence of bound substrate or product. In contrast, in the *Hs*UGP-UDP-Glc complex, the sidechain corresponding to Lys292 points toward UDP-Glc but is not oriented correctly to interact with it (54). In *Solanum tuberosum* UGP, a glutamine mutant of the residue corresponding to *An*UGP Lys292 displayed significantly decreased *V*_max_ values and increased *K*_*m*_ values for Glc-1-P and PPi (69). Similarly, the *Lm*UGP residue corresponding to *An*UGP Glu307 plays a primary role in binding of the Glc moiety, and consequently, its mutation caused a near-complete loss of activity and increased the *K*_*m*_ for Glc-1-P but not for UTP (60). Likewise, the homologous residue in *At*UGP (Glu271) was found to coordinate the Glc moiety (35). However, in the *Hs*UGP-UDP-Glc complex, Glu306 (corresponding to *An*UGP Glu307) appears to be located too far away to interact with the sugar ring (54). Similarly, *An*UGP Glu307 is placed 6 Å away from the nearest Glc atom when UDP-Glc is modeled in the binding pocket (Fig. 5D). It is therefore possible that the crucial role of these residues in Glc binding is limited to monomeric UGPs.

### Regulatory structure-function relationships in UGPs

#### (Inter)lock mechanism

In monomeric *Lm*UGP and *At*UGP, UTP binding triggers the closing of the NB-loop (Fig. 4) on the nucleotide portion of the substrate. In line with the sequential ordered mechanisms, the SB-loop is found in a closed conformation only once the sugar-binding site is occupied (35, 59, 60). In *At*UGP, these local conformational changes are coupled to a displacement of the entire CT β-helix domain toward the catalytic domain (35). In *Lm*UGP, the global conformational changes are even more pronounced and enable the formation of hydrogen bonds between SB-loop residues Glu251 and Arg443 (located in the CT β-helix domain of the same subunit), which are more than 11 Å apart in the *apo* state. This contact stabilizes the SB-loop in the closed conformation of the enzyme and was termed “lock mechanism” (60).

The structural rearrangements observed in the active site of octameric *Hs*UGP upon UDP-Glc binding resemble those in monomeric *Lm*UGP and *At*UGP, but with smaller amplitude, and are not coupled to significant overall movements of the domains (54). Instead, in the product-bound state of the octameric *Hs*UGP, the side-to-side dimer and octamer, respectively, are 6.3% and 14% more compact than in the *apo* form, and a mutual intermolecular interaction between SB-loop residues Arg287 (corresponding to *Lm*UGP “lock residue” Glu251) and Asp456 from the CT domain of its “side-to-side” neighboring subunit was observed (54). This interaction was absent or weaker in apo *Hs*UGP (36), suggesting that it is reinforced in substrate/product-bound states. Mutation of both involved residues in *Hs*UGP did not affect oligomerization but caused a dramatic drop in activity (Table S3D), confirming the functional importance of this interaction (54), which was therefore termed “interlock mechanism” in reference to *Lm*UGP. The two interlock residues are strictly or functionally co-conserved in octameric fungal and animal UGPs, but not in monomeric protozoan and plant UGPs (Fig. 4). In *An*UGP, the corresponding positions are occupied by Lys288 (SB-loop) and Asp462 (LβH domain), respectively, and could engage in the same type of interaction, although density for the sidechains in this region is weak and interaction cannot be unequivocally ascertained.

#### Latch mechanism

Another set of intermolecular interactions within UGP octamers, with the potential to influence enzymatic function, was proposed by Yu and Zheng: in *Hs*UGP, a so-called latch loop from one subunit is located between the SB-loop and a small adjacent loop (termed “309 loop”) of its side-to-side connected subunit (36). According to the authors, the latch loop of *Hs*UGP may sterically interfere with conformational changes associated with the catalytic cycle, and ultimately limit *Hs*UGP activity. The authors argue that in *Sc*UGP, this latch effect is not observed due to differences in the amino acid sequence of the latch loop, resulting in a higher activity of *Sc*UGP compared to *Hs*UGP. We hypothesize that at least the interaction between the latch- and 320-loops (the equivalent of the *Hs*UGP “309-loop”) is maintained in *An*UGP but dependent on conformational changes within the complex. Namely, we describe two major modes to this variability (Fig. 6; Video S2) that allow the UGP subunit to swing in orthogonal directions and change the interface with neighboring subunits. This changing interface imparts a dynamic nature to the latch mechanism, namely in *An*UGP, the latch-to-320-loop interface has the potential to be formed or broken depending on the swinging movement of individual UGP subunits in the octameric complex (Fig. 6; Video S2). Moreover, this interface is in proximity to the potential *An*UGP interlock residue Lys288 (Fig. 6G) opening up the possibility that the aforementioned interlock mechanism and the latch mechanism are intertwined and dependent on conformational changes in the octameric UGP. As Yu and Zheng demonstrated that mutations in the *Hs*UGP latch and 309-loops can affect the enzymatic activity of *Hs*UGP (Table S3C) (36), we propose that the dynamic nature of the interaction would similarly alter activity, potentially by stabilizing/destabilizing specific conformations of the UGP-Glc binding pocket.

### Role of the N-terminus

Across eukaryotic UGPs, the N-terminus shows the most diversity in terms of length and sequence, even between species as closely related as *A. fumigatus* and *A. nidulans* (Fig. 4). In crystal structures, this region forms a two-helix bundle, but typically the first 4 to 41 residues are poorly resolved and not traced. Our analysis, however, indicates that conformational changes in the monomer can lead to the partial ordering of the NT residues to make an interface, NT-to-β14/15-loop, with the neighboring monomer (Fig. 6H and Video S2). In line with its exposed position at the periphery of the octameric complex, the UGP NT domain has been proposed to be the subject of regulatory mechanisms (70, 71). In *S. cerevisiae*, changes in environmental and nutrient conditions cause PAS kinase to phosphorylate *Sc*UGP at Ser11 (*An*UGP Ser25), which targets the enzyme to the cell periphery, where its product UDP-Glc can be preferentially utilized for cell wall glucan synthesis (70, 71). *Hs*UGP exists in two isoforms encoded by the same gene, with the shorter isoform 2 being N-terminally truncated by 11 amino acids and predominantly expressed in the brain (72). This tissue-specific expression may imply that the two isoforms exhibit functional differences and/or different regulatory potential. For example, phosphorylation could serve as a means of differential regulation of the two isoforms, as an abundance of studies detected phosphorylation of Ser13 of *Hs*UGP isoform 1, but not of the corresponding residue Ser2 of *Hs*UGP isoform 2 (phosphosite.org entries “UGP2” and “UGP2 iso2,” respectively; accessed 10/28/2022). Mammalian UGPs are furthermore frequently phosphorylated at Tyr89, Tyr186, Tyr298, and Ser448 (*Hs*UGP isoform 1 numbering), which, except for *Hs*UGP Tyr186, are conserved in *An*UGP (Tyr101, Tyr299, and Ser454, respectively). While no phosphorylation of *An*UGP was observed in a recent phosphoproteomic study (personal communication), this does not exclude that under certain physiological conditions, phosphorylation or other types of posttranslational modifications may occur at the exposed NT domain or elsewhere in the protein and affect, for example, *An*UGP function, localization, or interaction with other factors.

In conclusion, the *An*UGP structure reveals extensive interconnectivity between the subunits, namely each subunit shares interfaces with four of the other seven subunits in the complex (Fig. 3K). There are relatively stable interfaces like the “end-to-end” and “side-to-side” interfaces (A–H and A–E; Fig. 3K), involving the LβH. At the same time, there are a dynamic set of interfaces (NT-to-β14/15-loop, latch-to-320-loop; Fig. 6G and H; Fig. 3K stars) whose formation is dependent on the swinging motion of the subunit within the octameric complex (Video S2). Interestingly, these two dynamic interfaces sit at the opposite ends of strand β15 and, thus, together, could influence *An*UGP activity via the 320-loop, which is close in primary sequence to the sugar-binding residues Asn329 and Asn331. The higher sequence divergence in these interfaces (Fig. 4) and their potential to allosterically influence enzymatic activity (i.e., interlock, 309-loop, and latch loop mutants; Fig. 7) could make these regions suitable targets for future development of allosteric UGP inhibitors that target sites located remotely from the conserved active site. The potential for selective allosteric inhibition of UGPs is evidenced by the identification of a small molecule targeting the monomeric L*m*UGP (37). Increasing our understanding of allosteric mechanisms through structural studies like those presented here is opening doors for rational allosteric inhibitor design (73).

## MATERIALS AND METHODS

### *An*UGP isolation and purification

Native *An*UGP (UniProt ID Q5I6D1; FungiDB ID AN9148) was co-purified from *A. nidulans* owing to its inherent ability to bind Ni-affinity columns (33). Specifically, *A. nidulans* was grown in yeast-extract-sucrose broth (2% w/v yeast extract, 6% w/v sucrose, pH 5.8) supplemented with Hunter’s trace elements (https://www.fgsc.net/methods/anidmed.html) at 30°C (150 rpm) for 4 d. The biomass was subsequently collected by filtration through Miracloth, dried by blotting with paper towels, and stored at −80°C. To lyse the cells, the pellet was first ground in a mortar (under liquid nitrogen), then the paste was resuspended in buffer A (50 mM Tris, 200 mM NaCl, 2 mM β-mercaptoethanol; pH 7.8) supplemented with DNAse and sonicated. The lysate was cleared by centrifugation (JA 25.50 rotor, 20,000 rpm, 20 min), filtered through a 0.22 µm syringe filter, and loaded to a HisTrap HP 1 mL column equilibrated in buffer A. The protein was eluted early in a buffer B gradient (50 mM Tris, 200 mM NaCl, 500 mM imidazole, 2 mM β-mercaptoethanol; pH = 7.8). The collected fractions were pooled, and a 12% SDS-PAGE gel showed the presence of a ~55 kDa protein and a larger ~130 kDa band. These bands were cut from the gel and analyzed by mass spectrometry (CIC bioGUNE Bilbao, Spain), which identified the 55 kDa protein as *An*UGP (UniProt ID Q5I6D1) and the larger band as pyruvate carboxylase. For cryoEM, the pooled fractions from the HisTrap column were loaded to a Superose 6 GL 10/300 equilibrated in buffer A (Fig. S1A and B), and a peak was observed in the elution profile around 14.5 mL which corresponds to an apparent molecular weight of 490 kDa (expected: 460 kDa for an *An*UGP homoctamer). The fractions corresponding to this peak were stored (−80°C) in buffer A at a concentration of 0.18 mg/mL. Initial screening at the CIC bioGUNE facility indicated that on the grid, the protein was of sufficient purity, monodispersed, and largely octameric, consistent with the oligomeric state of *An*UGP, strongly suggesting the co-isolated pyruvate carboxylase did not readily adhere to the grids during vitrification and/or is monomeric and not easily seen owing to its smaller size (Fig. S1C).

### MG phosphate assay

The enzymatic activity of *An*UGP was measured using the MG phosphate assay (74). *An*UGP catalyzes the condensation reaction between UTP and Glc-1-P to produce UDP-Glc and PPi, in the presence of the divalent metal cation Mg^2+^. This reaction is coupled to the conversion of PPi into orthophosphate (Pi), mediated by the inorganic pyrophosphatase from *Saccharomyces cerevisiae* (*Sc*PPase). The green complex formed between Pi, molybdate, and the MG dye is photometrically quantified. The assay reactions are as follows:


(1) $$\begin{array}{l} \alpha -Glc-1-P+UTP\to UDP-Glc+P_{2}O_{7}^{4-} \end{array}$$

(2) $$\begin{array}{l} P_{2}O_{7}^{4-}+H_{2}O\to 2PO_{4}^{3-}+2H^{+} \end{array}$$

(3) $$\begin{array}{l} H_{3}PO_{4}+12H_{2}MoO_{4}\to H_{3}PO_{4}(MoO_{3}{)}_{12}+12H_{2}O \end{array}$$

(4) $$\begin{array}{l} H_{3}PMo_{12}O_{40}+HMG^{2+}\to [MG+](H_{3}PO_{4}(MoO_{3}{)}_{12})+2H^{+} \end{array}$$

The reaction [1] is stopped by adding the chelating agent EDTA. The MG reaction [4] is stabilized by the addition of sodium citrate to avoid product precipitation. The samples contained 0.5 µg/mL of *An*UGP and 31 mM of inorganic pyrophosphatase (Sigma-Aldrich, St. Louis, Missouri), 0.25 mM NTPs (UTP, GTP, or ATP), 0.25 mM Glc-1-P, 50 mM Tris-HCl pH 7.5, 2 mM MgCl_2_, and 100 mM NaCl in a final volume of 60 µL. Reactions were incubated at 30°C and stopped at 25 min by the addition of 10 µL of 50 mM EDTA, pH 8.0. Afterward, 20 µL of molybdate solution containing 34 mM ammonium molybdate tetrahydrate in 4 N HCl (Fluka Chemie GmbH, Buchs, Switzerland) was added to the reaction mixture and incubated at 20°C for 3 min. Then, 60 µL of 1 mM MG solution (Sigma) was added to the mixture and incubated at 25°C for 5 min. Finally, the solution was stabilized by the addition of 60 µL of 170 mM sodium citrate (Sigma) and measured at 620 nm in a Spectra Max M2 plate reader (Molecular Devices, LLC, San Jose, CA). Measurements were performed in quintuplicate. *An*UGP was stored at 0.2 mg/mL in 50 mM Tris-HCl pH 7.8, 200 mM NaCl, and 2 mM β-mercaptoethanol at −80°C.

### Generation of recombinant strains

The DNA construct for the generation of the *A. nidulans* null mutant of *galF* was synthesized by fusing three PCR amplicons (75). First, a 1.5 Kb fragment corresponding to the promoter region of *galF* was amplified using oligonucleotides galF-PP1 (GTCCAAGCTGGCGCTCTGGC) and galF-PP2 (CATTGTGTGTGTTTTTGATGTGTTGTATGCG), and wild-type genomic DNA as template. Second, the selection marker *pyrG^Afum^* of *A. fumigatus* was amplified using oligonucleotides galF-SMP1 (CGCATACAACACATCAAAAACACACACAATGACCGGTCGCCTCAAACAATGCTCT) and galF-GFP2 (GCTTCAGTGGCCAATTAATGCTCGAGGTCTGAGAGGAGGCACTGATGCG), using plasmidic DNA as a template (75). Third, a 1.5 Kb fragment corresponding to the 3´-UTR region of *galF* was amplified with oligonucleotides galF-GSP3 (CTCGAGCATTAATTGGCCACTGAAGC) and galF-GSP4 (GAGGGAACACAGCACGTGCC), and genomic DNA as template. The fusion-PCR reaction was carried out with oligonucleotides galF-PP1 and galF-GSP4, and the correct generation of the DNA construct was verified by agarose electrophoresis. A high-fidelity DNA polymerase was used in these PCR reactions (PrimeStar, Takara Bio Inc, Kusatsu, Shiga, Japan). Protoplasts of *A. nidulans* strain TN02A3 (genotype: *pyrG89; argB2; pyroA4,* Δ*nkuA::argB; veA1*) (26) were obtained and transformed based on the procedures developed by Tilburn and colleagues, as well as Szewczyk and colleagues (76, 77). Transformants were selected on a regeneration medium (RMM), a minimal medium (AMM; see below) supplemented with 1 M sucrose. This medium lacked uridine and uracil (the nutrients associated to the marker *pyrG^Afum^*) to enable growth of only transformants. In general, strains were cultivated in AMM (78, 79) adequately supplemented according to their genetic markers. Glc (2%) or Gal (1%) and ammonium tartrate (5 mM) were used as sources of carbon and nitrogen, respectively.

### Diagnostic PCR reactions

Adequately supplemented liquid AMM was inoculated with 10^6^ conidia mL^−1^. Strains were cultured for 18 h at 37°C and 150 rpm. Mycelia were filtered using Miracloth paper and lyophilized overnight. Samples were then homogenized with 5 mm beads (Bullet Blender CE; Next Advance, Inc, Troy, NY), and DNA fractions were extracted using the phenol:chloroform:isoamyl alcohol (25:24:1) procedure (80). To verify the correct integration of the DNA constructs and the homokaryotic, heterokaryotic, or diploid nature of the recombinant strains generated, diagnostic PCR reactions were run using genomic DNA samples as template and three oligonucleotide pairs. A first set of PCR reactions was run using primers galF-sPP1 (GTGGCTCCCTGACAGGCTGG) and galF-sGSP4 (CATAGCTCACTATGCTGCTGCTGG). These two oligonucleotides correspond to 5´- and 3´-UTR regions located upstream and downstream of primers galF-PP1 and galF-GSP4, respectively, which were used to generate by fusion-PCR the transformation cassette (see previous section). The amplicons that resulted from wild-type or transformant DNA samples could not be differentiated due to the similar length of *galF* (1.98 Kb) and the *pyrG^Afum^* fragment (1.90 Kb) and, thus, this reaction was used as control (not shown). A new set of PCR reactions was then run with primer pairs galF-sPP1 (located approximately 125 nucleotides upstream of galF-PP1) and galF-insideRP (GCTCGATCTGACGGACGGAC), or galF-sPP1 and pyrG-insideRP (GCCCGTAGCCAGCGATCC).

### Sample vitrification and electron microscopy

Screening the sample for cryoEM suitability was done using facilities at the CIC bioGUNE (Bilbao, Spain) and initially imaged at the CNB-CSIC (Madrid, Spain; Instruct PID 3796). *An*UGP in buffer A (0.18 mg/mL) was vitrified using a Vitrobot (FEI) onto Quantifiol R1.2/1.3 (Cu 300) grids. Automated high-resolution data acquisition (EPU software, V2.8.1, Thermo Fisher Scientific, Waltham, MA) was performed at NeCEN (Netherlands Centre for Electron Nanoscopy, Instruct -PID14910) with a Titan Krios microscope (Thermo Fisher Scientific) at 300 kV equipped with a K3 direct electron detector (Table S1). Eight thousand one hundred thirty-five movies were collected, with each movie containing 50 frames at a pixel size of 0.545 Å (super-resolution) and a total dose of 60 e^-^/Å^2^. More detailed imaging conditions are presented in Table S1.

### Image processing and structure determination

All image processing steps were performed within the CryoSPARC and CryoSPARC Live software package (81) apart from particle picking, which was done in RELION-3.1 (82) using crYOLO (83). Movies were imported to CryoSPARC, patch motion corrected, while binning by a factor of 2 to the physical pixel size (1.09 Å/pixel), and the contrast transfer function estimated. Subsequently, the patch-aligned micrographs were imported into RELION and particle picking was performed with crYOLO, yielding a total of 451,301 projection images. The particle coordinates were reimported into CryoSPARC and micrographs were manually curated based on estimated resolution, defocus values, and motion parameters. The particles were windowed out and downsampled to a box size of 128 pixels × 128 pixels (2.18 Å/pixel) for initial cleaning (Fig. S3A and B). Several rounds of 2D classification were performed and in each round, the best classes resembling an octameric *An*UGP were selected, finally corresponding to 204,062 particles. The selected 2D classes (Fig. S3C) were rebalanced, and these particles (81,156) were used for *ab initio* reconstruction (one class; Fig. S3D). Subsequently, the full set of particles (204,062) were refined, using C1 symmetry, to generate a 4.9Å cryoEM map (Fig. S3E). Classification at this stage did not indicate contamination with other particles like the pyruvate carboxylase. Phenix.map_symmetry (84) showed that the C1 map displayed D4 symmetry and, therefore, a non-uniform refinement was run while applying D4 symmetry resulting in a 4.5 Å structure (Nyquist is 4.38 Å). Note that refinements employing lower-order symmetry did not improve the interpretability or resolution of the map as compared to D4. Because the D4 refinements were converging to near the Nyquist frequency, the particles were re-extracted and recentered to their full pixel size (256 pixels × 256 pixels, 1.09 Å/px). Heterogenous refinement (three classes) was used to clean out poorly aligning, junky, or broken particles. The best two classes were joined and used in a non-uniform refinement to generate a cryoEM map at 3.98 Å resolution (Fig. S3F and G). This map showed obvious signs of flexibility in the subunits that break the D4 symmetry, so we employed symmetry expansion, followed by signal subtraction and a local restrained refinement (5° gaussian prior over rotation and 3 Å gaussian prior over shifts) to generate a map for a single *An*UGP monomer (Fig. S3H and I). Sharpening was performed with DeepEMhancer using the non-uniform refinement half maps generated in cryoSPARC (85). Local resolution was estimated with CryoSPARC using the non-uniform refinement half maps (86). Conformational variability was performed by downsampling the symmetry-expanded particles (before subtracting and local alignment) to a box size of 128 pixels × 128 pixels (2.18 Å/px). 3D classification without alignment (10 classes) was first used to clean the data set further and remove poorly aligned or broken particles. The best particle images (nine classes) were joined and subjected to 3DVA in CryoSPARC, solving for 10 PCs. The first two components yielded easily interpretable conformation changes and therefore were used to generate intermediate maps for analysis.

### Model building

The AlphaFold tool in ChimeraX (87) was used to generate an initial model from the *An*UGP sequence (UniProt ID Q5I6D1). The resulting model was imported into Isolde (88) and refined using interactive molecular-dynamics flexible fitting into the unsharpened D4 map, the sharpened D4 map, and the locally refined sharpened map. Distance and torsion restraints were applied using the AlphaFold model, the *Sc*UGP model, and the *Af*UGP model as references. The resulting model was subsequently subjected to Real Space Refinement in Phenix (89) using the parameters output from Isolde (isolde write phenixRsrInput) and the sharpened D4 non-uniform refinement map. Statistics for this model were calculated using phenix.validation_cryoEM and the sharpened D4 non-uniform refinement map and corresponding half maps (Table S2).

## Data Availability

The cryoEM movies, cryoEM maps, and atomic coordinates have been deposited with the Protein Data Bank (PDB), EMDB, and EMPIAR accession codes 8C0B , 16357, and EMPIAR-11471. Data are available from the corresponding authors upon reasonable request.

## SUPPLEMENTAL MATERIAL

Supplemental material mbio.00414-23-s0001.pdf Supplemental figures and tables.

Video S1 mbio.00414-23-s0002.mov AnUGP cryoEM map (D4) and model. The sharpened AnUGP D4 cryoEM map is shown with the model superimposed (cartoon representation). The map and model are colored according to the domain structure defined in Figure 2A. The grey regions of the map are density that is unmodeled (e.g., NT residues).

Video S2 mbio.00414-23-s0003.mp4 Conformational flexibility in AnUGP. The movie shows the sequence of intermediate reconstructions (intermediates 1-10) for each principal component from two orthogonal views. The analyzed monomer is colored according to the domain structure defined in Figure 2A, while the remaining seven monomers are grey.

ASM does not own the copyrights to Supplemental Material that may be linked to, or accessed through, an article. The authors have granted ASM a non-exclusive, world-wide license to publish the Supplemental Material files. Please contact the corresponding author directly for reuse.
