# Supplementary material for: CryoEM analysis of the essential native UDP-glucose pyrophosphorylase from Aspergillus nidulans reveals key conformations for activity regulation and function
Source: mBio. 2023 Jul 6;14(4):e00414-23. doi: 10.1128/mbio.00414-23 (PMC10470519; doi:10.1128/mbio.00414-23)
Supplement: Supplemental material — Supplemental figures and tables. [file mbio.00414-23-s0001.pdf]

SUPPORTING INFORMATION

CryoEM analysis of the essential native UDP-glucose pyrophosphorylase from *Aspergillus nidulans* reveals key conformations for activity regulation and function

Xu Han,[1,2,#] Cecilia D'Angelo,[2,3,#], Ainara Otamendi,[4] Javier O. Cifuente,[2,3] Elisa de Astigarraga,[1,2] Borja Ochoa-Lizarralde,[1,2] Martin Grininger,[5] Françoise H. Routier,[6] Marcelo E. Guerin[2,3,7], Jana Fuehring,[6,*] Oier Etxebeste,[4,*] Sean R. Connell.[1,2,7*]

[1] Structural Biology of Cellular Machines Laboratory, Biocruces Bizkaia Health Research Institute, Cruces University Hospital, 48903 Barakaldo, Bizkaia, Spain.
[2] Center for Cooperative Research in Biosciences (CIC bioGUNE), Basque Research and Technology Alliance (BRTA), Bizkaia Technology Park, Building 801A, 48160 Derio, Spain.
[3] Structural Glycobiology Laboratory, Biocruces Bizkaia Health Research Institute, Cruces University Hospital, 48903 Barakaldo, Bizkaia, Spain.
[4] Laboratory of Biology, Department of Applied Chemistry, Faculty of Chemistry, University of the Basque Country, UPV/EHU, Manuel de Lardizabal 3, 20018 San Sebastian, Spain.
[5] Institute of Organic Chemistry and Chemical Biology, Buchmann Institute for Molecular Life Sciences, Goethe University Frankfurt, Frankfurt am Main, Germany.
[6] Institute for Clinical Biochemistry, OE4340, Hannover Medical School, Carl-Neuberg-Strasse 1, 30625 Hannover, Germany.
[7] Ikerbasque, Basque Foundation for Science, 48009 Bilbao, Spain.

[#]These authors contributed equally

Table of Contents

1. Supplementary Tables

Supplemental Table S1: EM Data Collection and Image Processing.

Supplemental Table S2: EM Model and Refinement Statistics.

Supplemental Table S3: Octameric UGP mutagenesis studies

2. Supplementary Figures

Supplemental Fig. S1: *An*UGP isolation and purification.

Supplemental Fig. S2: *AN9148/galF* is an essential gene.

Supplemental Fig. S3: Cryo-EM Processing.

Supplemental Fig. S4: Comparison of structural homologues of *An*UGP.

Supplemental Fig. S5: Octameric structures of UGPs.

Supplemental Fig. S6: Proposed catalytic mechanism of *An*UGP.

Supplemental Fig. S7: Evolutionary analyses of UGPs.

3. Supplementary Movies

Supplemental Movie S1: ***An*UGP cryoEM map (D4) and model.** The sharpened *An*UGP D4 cryoEM map is shown with the model superimposed (cartoon representation). The map and model are colored according to the domain structure defined in **Figure 2**. The grey regions of the map are density that is unmodeled (e.g., NT residues).

Supplemental Movie S2: **Conformational flexibility in *An*UGP.** The movie shows the sequence of intermediate reconstructions (intermediates 1-10) for each principal component from two orthogonal views. The analyzed monomer is colored according to the domain structure defined in **Figure 2**, while the remaining seven monomers are grey.

1. Supplementary Tables

Supplemental Table S1. EM Data Collection and Image Processing.

Data Collection	
Sample	**EMD-16357**<br>**EMPIAR-11471**<br>***An*UGP**
Facility (ID)	NeCEN (PID 14910)
Microscope	Titan Krios
Camera	K3 (counting)
Data Collection Software	EPU (Thermo Fisher)
Voltage (kV)	300
Calibrated Pixel Size (Å)	1.09
Total Exposure (e^-/ $Å^2$)	60
Total Exposure Time (s)	8
Number of Frames	50
Defocus Range (µm)	-0.5 to -3.0
**Image Processing**	
Motion Correction Software	CryoSPARC
CTF estimation software	CryoSPARC
Particle Section	crYOLO
Micrographs Collected	8135
Particles Selected	451301
Classification and Refinement Software	CryoSPARC
**Model Building**	
Visualisation Software	ChimeraX
Refinement Software	Phenix, Isolde

Supplemental Table S2. EM Model and Refinement Statistics.

**Model**		
	PDB 8C0B	
Composition (#)		
Chains	8	
Atoms	29816 (Hydrogens: 0)	
Residues	Protein: 3768 Nucleotide: 0	
Water	0	
Ligands	0	
Bonds (RMSD)		
Length (Å) (# > 4σ)	0.003 (0)	
Angles (°) (# > 4σ)	0.787 (0)	
MolProbity score	1.47	
Clash score	3.97	
Ramachandran plot (%)		
Outliers	0.22	
Allowed	3.92	
Favoured	95.86	
Rama-Z (Ramachandran plot Z-score, RMSD)		
whole (N = 3720)	-0.95 (0.13)	
helix (N = 1040)	-1.06 (0.14)	
sheet (N = 704)	0.08 (0.21)	
loop (N = 1976)	-0.53 (0.14)	
Rotamer outliers (%)	0.51	
Cβ≤ outliers (%)	0	
Peptide plane (%)		
Cis proline/general	0.0/0.0	
Twisted proline/general	0.0/0.0	
CaBLAM outliers (%)	0.90	
ADP (B-factors)		
Iso/Aniso (#)	29816/0	
min/max/mean		
Protein	55.70/255.06/137.66	
Nucleotide	---	
Ligand	---	
Water	---	
Occupancy		
Mean	1	
occ = 1 (%)	100	
0 < occ < 1 (%)	0	
occ > 1 (%)	0	
**Data**		
Box		
Lengths (Å)	153.69, 152.60, 138.43	
Angles (°)	90.00, 90.00, 90.00	
Supplied Resolution (Å)	4	
Resolution Estimates (Å)	Masked	Unmasked
d FSC (half maps; 0.143)	4	4.1
d 99 (full/half1/half2)	2.8/4.5/4.6	2.8/3.9/3.9
d model	2.1	2.1
d FSC model (0/0.143/0.5)	1.8/2.2/4.4	1.8/2.2/4.4
Map min/max/mean	-0.00/2.06/0.02	
**Model vs. Data**		
CC (mask)	0.68	
CC (box)	0.69	
CC (peaks)	0.66	
CC (volume)	0.68	
Mean CC for ligands	---	

Supplemental Table S3. Summary of Octameric UGP mutation studies

Active site mutagenesis [1]						
**AnUGP**	**HsUGP***	**HsUGP#**	**Mutant**	**Activity (% of wt.)**		**Oligomerization state**
				**Forward**	**Reverse**	
L121	L113	L102	L113G	insoluble	insoluble	insoluble
G123	G115	G104	G115D	0.0044 ± 0.0003	not determined	octamer
G124	G116	G105	G116A	0.0676 ± 0.0113	not determined	octamer
K135	K127	K116	K127A	0.1500 ± 0.0092	not determined	octamer
G227	G222	G211	G222A	insoluble	insoluble	insoluble
H228	H223	H212	H223L	insoluble	insoluble	insoluble
N256	N251	N240	N251L	0.0111 ± 0.0009	not determined	octamer
D258	D253	D242	D253L	0.0632 ± 0.0043	not determined	octamer
N329	N328	N317	N328L	insoluble	insoluble	insoluble
K403	K396	K385	K396A	0.0413 ± 0.0028	not determined	octamer
"309 loop", "latch loop", oligomerization domain mutagenesis [3]						
**AnUGP**	**HsUGP***	**HsUGP#**	**Mutant**	**Activity (% of wt.)**		**Oligomerization state**
				**Forward**	**Reverse**	
S321/K323	S320/S322	S309/S311	S309N/S311R	*not determined*	83,64 ± 5,97	octamer
V424/M425	T417/M418	T406/M407	T406K/M407L	*not determined*	130,39 ± 12,21	octamer
V424-V433	T417-V427	T406-V416	hUGP Y-latch[4]	*not determined*	148,05 ± 8,83	octamer
N428	K421	K410	K410S	*not determined*	256,62 ± 13,25	octamer
(absent)	E423	E412	E412Q	*not determined*	119,48 ± 6,49	octamer
(absent)	E423	E412	E412D	*not determined*	175,58 ± 3,38	octamer
(absent)	E423	E412	E412K	*not determined*	22,08 ± 0,78	octamer
G431/G432	P425/T426	P414/T415	P414G/T415P	*not determined*	86,49 ± 2,86	octamer
V433	V427	V416	V416N	*not determined*	129,09 ± 7,79	octamer
S508/L509	N502/L503	N491/L492	N491P/L492E	*not determined*	174,81 ± 9,87	monomer
Oligomerization domain mutagenesis [2]						
**AnUGP**	**HsUGP***	**HsUGP#**	**Mutant**	**Activity (% of wt.)**		**Oligomerization state**
				**Forward**	**Reverse**	
H463	H457	H446	H446S	76.5 ± 5.60	71.68 ± 5.51	octamer
T465	T459	T448	T448K	1.94 ± 0.23	3.24 ± 0.15	octamer
I483	I477	I466	I466T	2.10 ± 0.15	8.07 ± 0.24	octamer
V485	I479	I468	I468K	97.45 ± 5.50	67.90 ± 5.35	octamer
V504	I498	I487	I487D	5.34 ± 0.38	8.48 ± 0.75	tetramer
S508	N502	N491	N491P	94.63 ± 9.79	55.62 ± 5.48	octamer/dimer
L509	L503	L492	L492E	131.86 ± 5.24	99.93 ± 2.12	octamer/dimer
H514	H508	H497	H497A	4.30 ± 0.11	72.24 ± 5.40	octamer
S508/L509	N502/L503	N491/L492	N491P/L492E	8.15 ± 0.27	15.33 ± 0.43	dimer
G507-H514	G491-H508	G490-H497	Δ490-497	5.06 ± 0.29	11.04 ± 0.82	dimer

Supplemental Table S3. Continued.

"Interlock" mutagenesis [1]						
**AnUGP**	**HsUGP***	**HsUGP#**	**Mutant**	**Activity (% of wt.)**		**Oligomerization state**
				**Forward**	**Reverse**	
K288	R287	R298	R287E	6.45 ± 0.16	8.44 ± 0.57	octamer
K288	R287	R298	R287L	4.35 ± 0.04	6.86 ± 0.47	octamer
D462	D456	D467	D456K	0.80 ± 0.05	0.88 ± 0.03	octamer

[*] *Hs*UGP long isoform (508 AA); [#] *Hs*UGP short isoform (497 AA), [1] Führing et al. 2015; [2] Führing et al 2013; [3] Yu & Zheng 2012 (1–3).
[4] *Hs*UGP with latch loop of *S. cerevisiae* UGP.

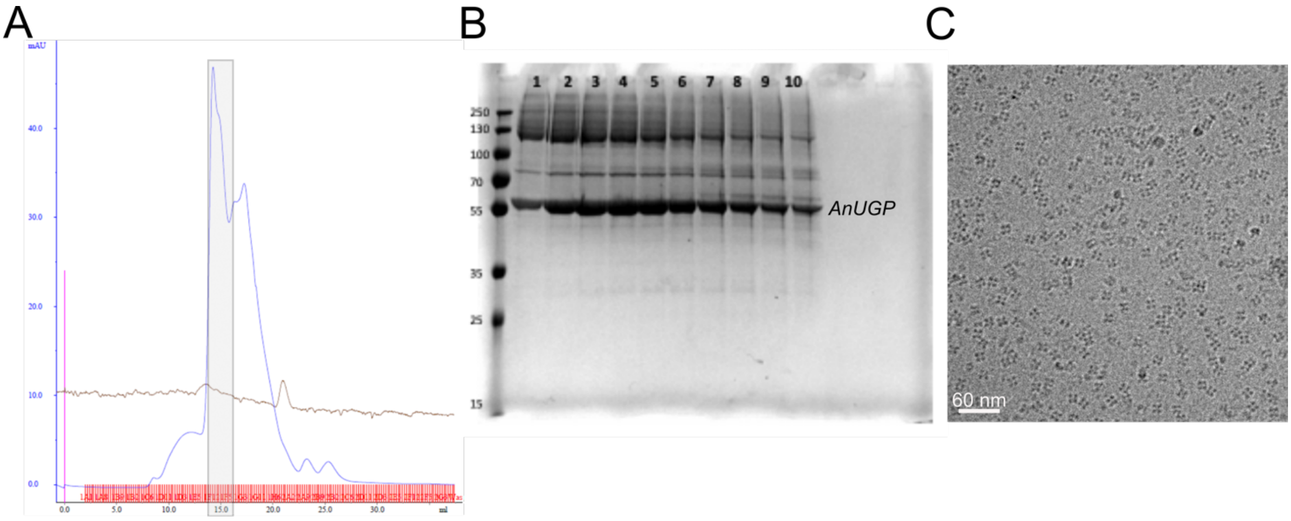

Supplemental Fig. S1. *An*UGP isolation and purification. (**A**) The elution profile of *An*UGP from a Superose 6 10/300 column. The grey transparent box indicates the fraction range shown in panel B. (**B**) Fractions from the Superose 6 10/300 column analyzed by 12 % Tris-Glycine SDS-PAGE. Mass spectrometry confirmed the lower band is *An*UGP while the upper ~130 kDa band is *A. nidulans* pyruvate carboxylase (**C**) CryoEM micrograph from the initial screening of the sample, which shows a monodisperse octameric particle consistent with *An*UGP.

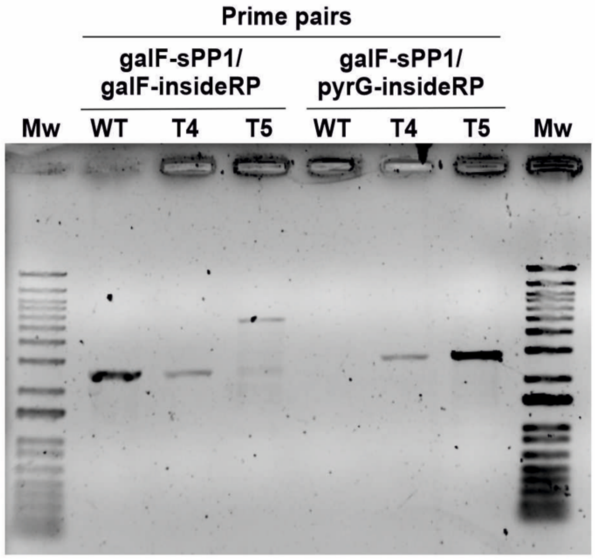

Supplemental Fig. S2. ***AN9148/galF* is an essential gene.** Agarose electrophoresis for the PCR products obtained when genomic DNA of diploids T4 and T5, or that of the wild-type strain, were used as templates, and oligonucleotide pairs galF-sPP1/galF-insideRP or galF-sPP1/pyrG-insideRP as primers. Oligonucleotide galF-sPP1 is located approximately 125 nucleotides upstream of galF-PP1, which was used to generate by fusion-PCR the transformation cassette. Thus, the use of galF-sPP1 allows confirmation of the integration of the transformation cassette at the *locus galF*. Results confirm the presence of both the parental *galF* allele and the selection marker $pyrG^{Afum}$ in the genomes of diploids T4 and T5.

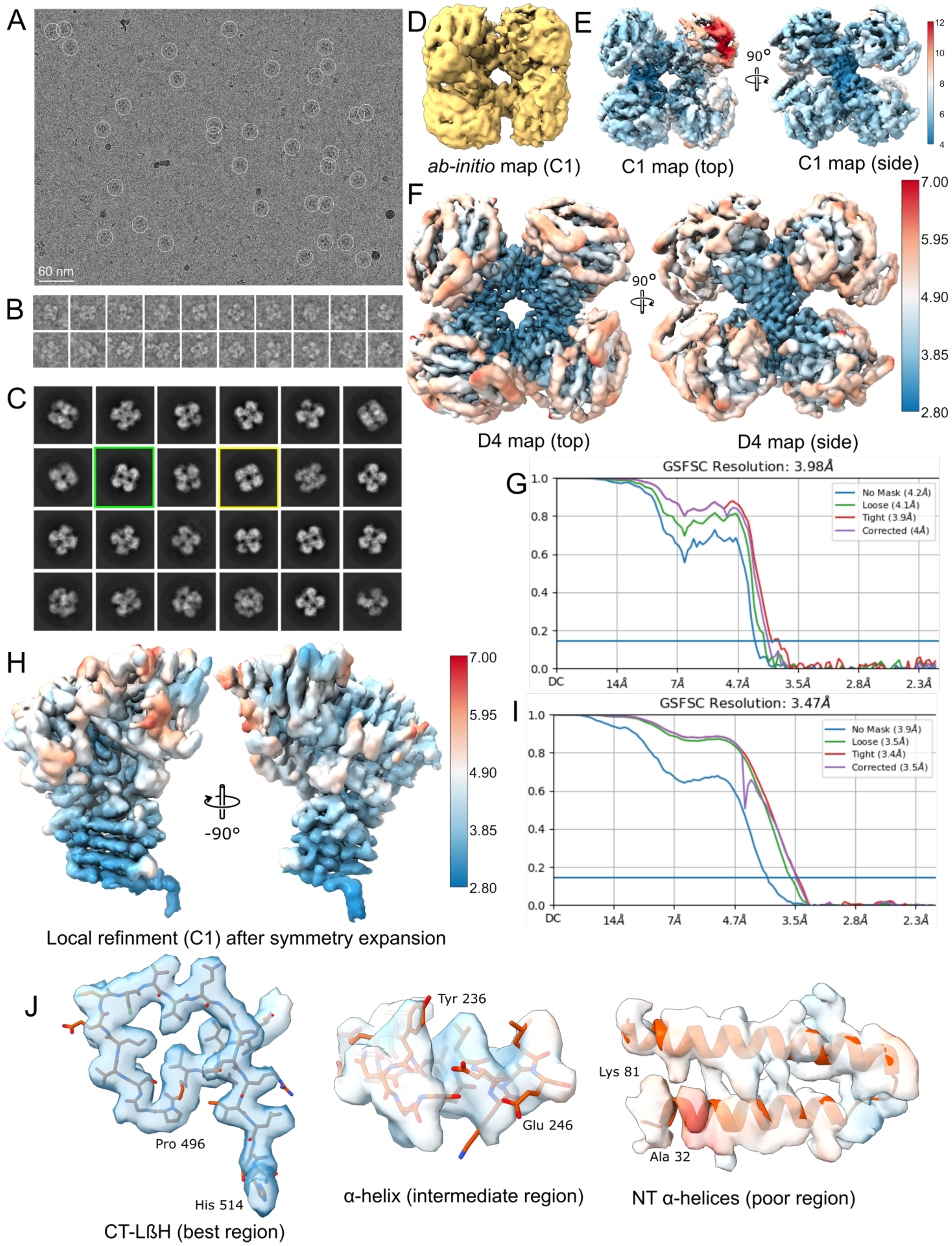
A
60 nm
B
C
D
ab-initio map (C1)
E
C1 map (top)
90°
C1 map (side)
F
90°
D4 map (top)
D4 map (side)
7.00
5.95
4.90
3.85
2.80
G
GSFSC Resolution: 3.98Å
No Mask (4.2Å)
Loose (4.1Å)
Tight (3.9Å)
Corrected (4Å)
DC
14Å
7Å
4.7Å
3.5Å
2.8Å
2.3Å
H
-90°
Local refinment (C1) after symmetry expansion
I
GSFSC Resolution: 3.47Å
No Mask (3.9Å)
Loose (3.5Å)
Tight (3.4Å)
Corrected (3.5Å)
J
Pro 496
His 514
CT-LßH (best region)
Tyr 236
Glu 246
α-helix (intermediate region)
Lys 81
Ala 32
NT α-helices (poor region)

Supplemental Fig. S3. Cryo-EM Processing. (**A**) Representative cryoEM micrograph. (**B**) Representative extracted particles (280 Å x 280 Å box). (**C**) Representative 2D Classes. A typical top view is boxed in yellow, and a side view in green. (**D**) The unbiased *ab-initio* cryoEM map in C1 (280 Å x 280 Å box) (**E**) The final sharpened (4) cryoEM map, which was refined with C1 symmetry (256x256 box; 1.09 Å/px; resolution is 4.81Å). This map is shown from the top and side. The map is colored according to local resolution as calculated with BlocRes (5) in CryoSPARC using the unsharpened refinement half maps. The central core formed by LßH has a local resolution of around 4 Å. (**F**) The final sharpened cryoEM map, refined with D4 symmetry (256x256 box; 1.09 Å/px; resolution is 3.98Å), seen from the top and side and colored according to local resolution. The LßH has a local resolution of around 3.2 Å. (**G**) The FSC curve for the final D4 map. The intermediate dip in the FSC curve, is often seen with membrane proteins and disorder in the micelle region of the map. Here we hypothesize that this dip corresponds to the lower order in the N- and central domains resulting from their independent movement, which breaks the D4 symmetry. (**H**) The sharpened cryoEM map for single subunit (subunit A) after symmetry expansion and local refinement (256x256 box; 1.09 Å/px; resolution is 3.49Å). The map is colored according to local resolution and masked to remove density corresponding to neighboring subunits. (**I**) The FSC curve for the locally refined subunit map. The intermediate dip is marginally improved in agreement with it, representing flexibility between the NT, central, and CT domains that the local refinement can partially overcome. (**J**) Selected regions of the sharpened map are shown to illustrate the local map and model quality. The map is colored according to the resolution key in panel H.

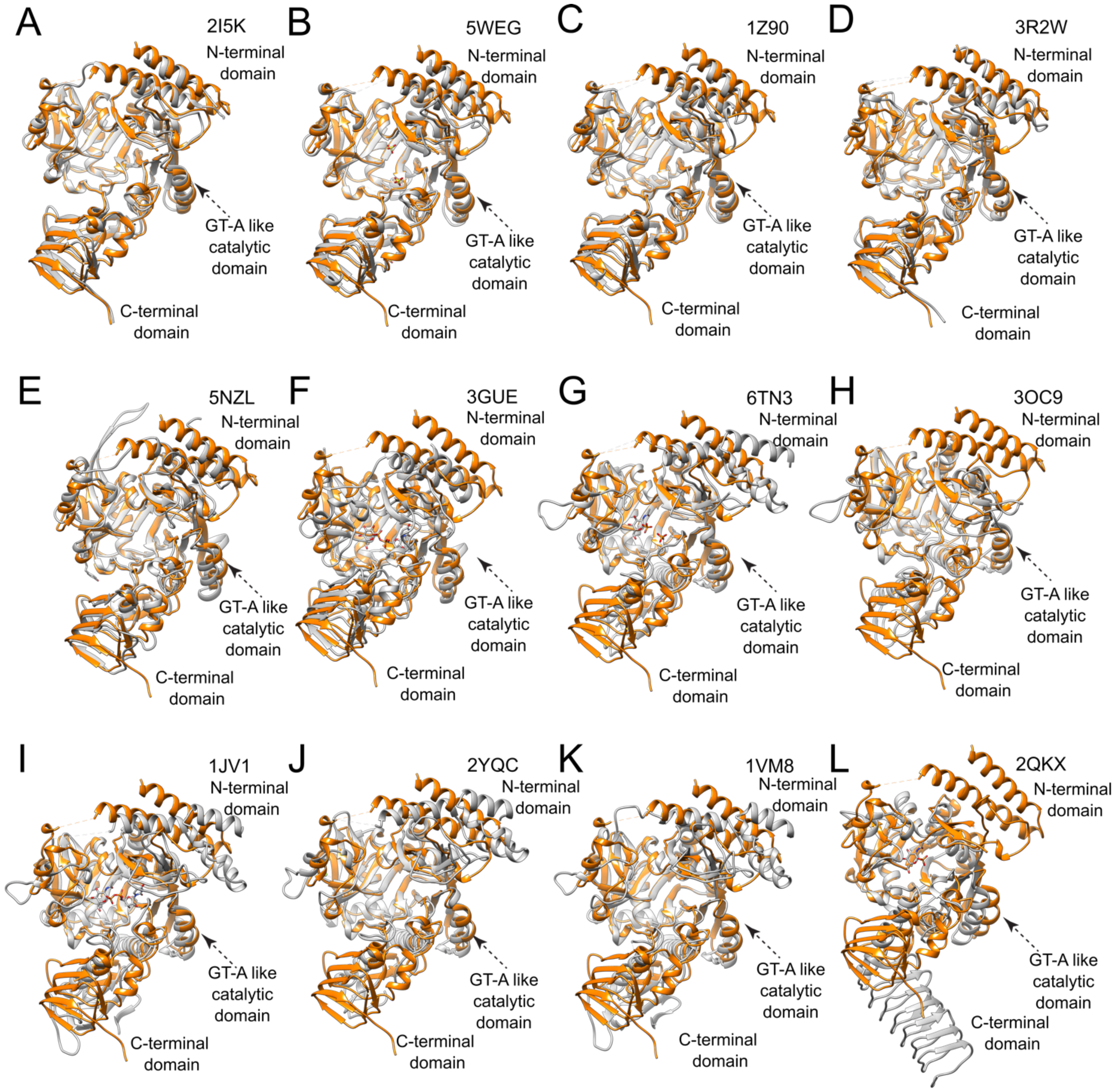

Supplemental Fig. S4. Comparison of structural homologues of *An*UGP. Structural comparison of an *An*UGP subunit (orange) and a protomer of the following structural homologues (grey): (**A**) UGP from *Saccharomyces cerevisiae* (*Sc*UGP; PDB code 2I5K; Z-score of 46.7; r.m.s.d. value of 1.5 Å for 462 aligned residues, 68% identity; (6)); (**B**) UGP from *Saccharum hybrid cultivar* SP80-3280 (*Sh*UGP; PDB code 5WEG; Z-score of 44.5; r.m.s.d. value of 1.5 Å for 440 aligned residues; 57% identity; (7)); (**C**) UGP from *Arabidopsis thaliana* (*At*UGP; PDB code 1Z90; Z-score of 44.4; r.m.s.d. value of 1.7 Å for 444 aligned residues, 56% identity; (8)); (**D**) UGP from *Homo sapiens* (*Hs*UGP; PDB code 3R2W; Z-score of 44.3; r.m.s.d. value of 1.7 Å for 455 aligned residues, 57% identity; (3)); (**E**) UGP from *Leishmania major* (*Lm*UGP; PDB code 5NZL; Z-score of 37.7; r.m.s.d. value of 2.3 Å for 441 aligned residues, 37% identity; (9)); (**F**) UGP from *Trypanosoma brucei* (*Tb*UGP; PDB code 3GUE; Z-score of 36.3; r.m.s.d. value of 3.4 Å for 419 aligned residues, 39% identity; (10)); (**G**) UDP-GlcNAc pyrophosphorylase (UGlcNAcP) from *Aspergillus fumigatus* (*Af*UGlcNAcP; PDB code 6TN3; Z-score of 31.2; r.m.s.d. value of 3.1 Å for 339 aligned residues, 20% identity; (11)); (**H**) UGlcNAcP from *Entamoeba histolytica* (*Eh*UGlcNAcP; PDB code 3OC9; Z-score of 30.2; r.m.s.d. value of 3.0 Å for 316 aligned residues, 18% identity; (12)); (**I**) UGlcNAcP from *Homo sapiens* (*Hs*UGlcNAcP; PDB code 1JV1; Z-score of 29.2; r.m.s.d. value of 3.2 Å for 350 aligned residues, 19% identity; (13); (**J**) UGlcNAcP from *Candida albicans* (*Ca*UGlcNAcP; PDB code 2YQC; Z-score of 29.0; r.m.s.d. value of 3.3 Å for 353 aligned residues, 18% identity; (14)); (**K**) UGlcNAcP from *Mus musculus* (*Mm*UGlcNAcP; PDB code 1VM8; Z-score of 28.7; r.m.s.d. value of 3.4 Å for 355 aligned residues, 18% identity); (**L**) bifunctional GlcN-1-P acetyltransferase / UDP-GlcNAc pyrophosphorylase GlmU from

Mycobacterium tuberculosis (*Mt*GlmU; PDB code 2QKX; Z-score of 18.2; r.m.s.d. value of 7.4 Å for 272 aligned residues, 15% identity; (15)).

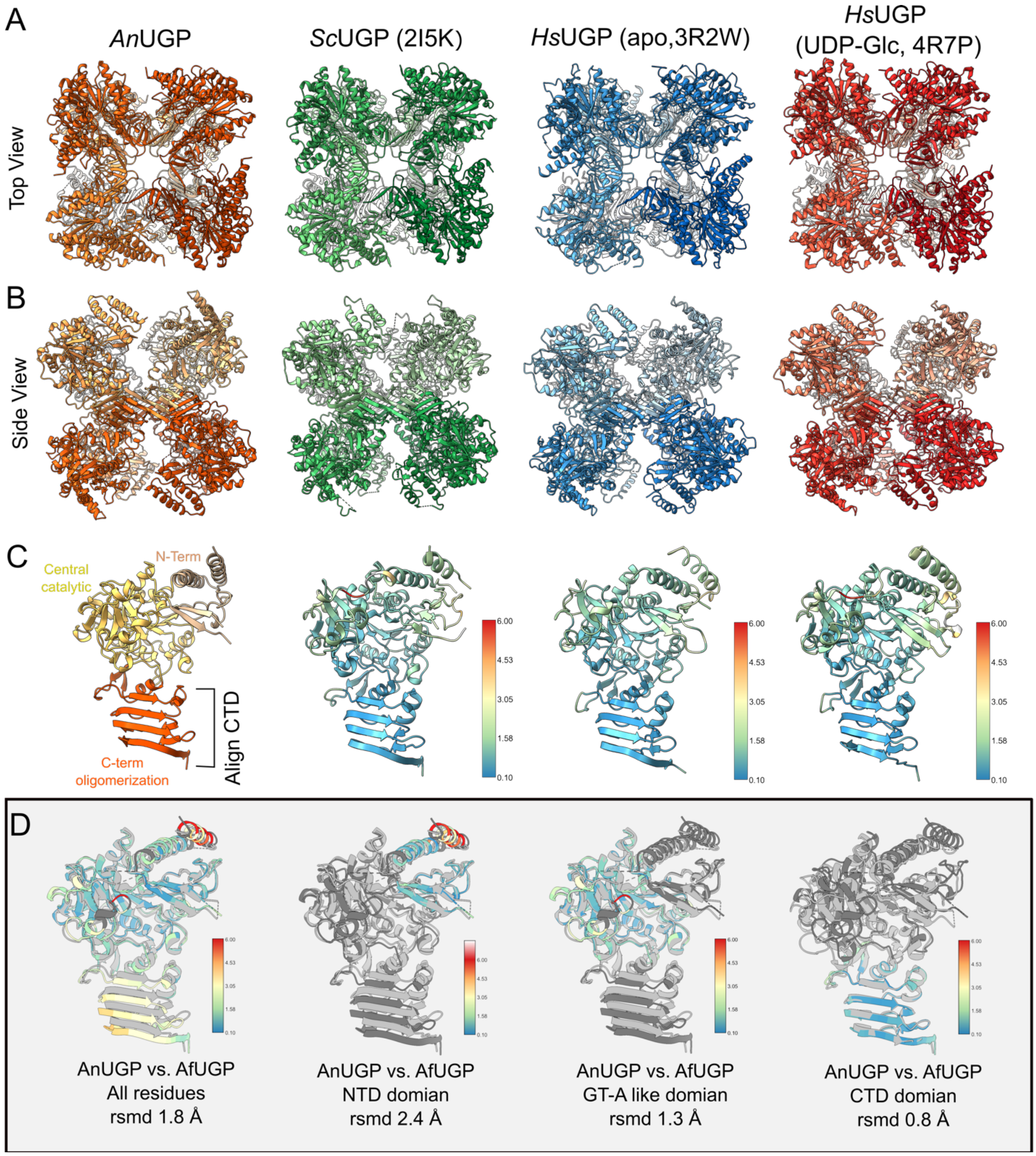

Supplemental Fig. S5. Octameric structures of UGPs. The structures of closely related Human (PDBID 3R2W and 4R7P) and *S. cerevisiae* (2I5K) UGP alongside *A. nidulans* *An*UGP are shown from the top (**A**) and side (**B**). To highlight conformational flexibility in the domains, the UGPs have been aligned on the C-terminal oligomerization domain and colored according to the r.m.s.d. of homologous residues after the alignment (**C**). *An*UGP is colored according to domains. (**D**) A comparison between *An*UGP and the crystal structure of the closely related *A. fumigatus* UGP (*Af*UGP; PDB 7ppr). To show the similarity between the fold of the monomeric unit from *An*UGP and *Af*UGP they have been aligned using all residues or residues corresponding to the NTD, central GT-A-like domain, and CTD. In each panel, the *Af*UGP is shown in light grey, while the AnUGP is colored according to RSMD. When coloring by RSMD, only the residues used for alignment are colored, with the remaining residues shown in dark grey. Generally, the similarity of the two structures increases from the NTD to the CTD, likely reflecting the decreased flexibility and corresponding improved local resolution (**Supplemental Figure 3**) of the CTD in the cryoEM map.

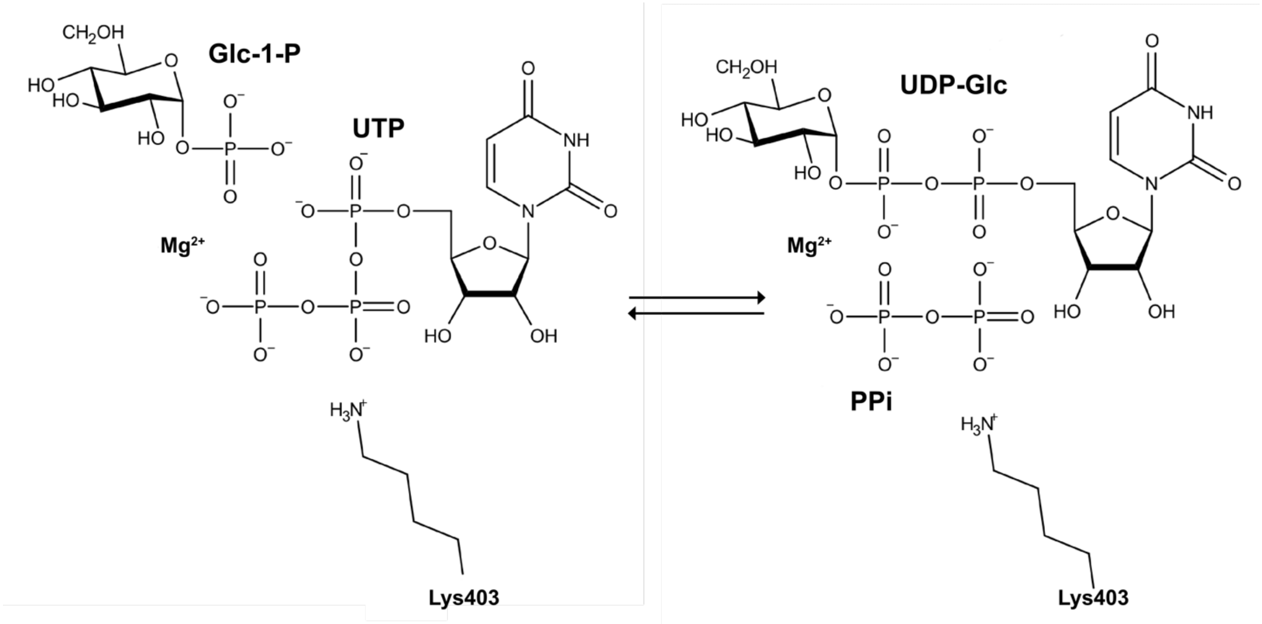

Supplemental Fig. S6. Proposed catalytic mechanism of *An*UGP. The formation of the nucleotide activated donor UDP-Glc occurs as a condensation reaction between UTP and Glc-1-P. Specifically, the oxygen on the sugar PO_4 acts as a nucleophile attacking the α-PO_4 group of the nucleoside triphosphate leading to the liberation of pyrophosphate (PPi). The reaction is held in the presence of cation Mg^{2+} (magnesium-dependent activity), which minimizes the charge repulsion between anionic phosphorous groups and proved to induce nucleophile activation in other nucleotidyltransferases (16). In addition, Lys403 likely polarizes these groups, increasing the nucleophilic nature of the oxygen attacking the phosphorus atom. In nature, the reaction is pulled forward by the subsequent hydrolysis of PPi by inorganic pyrophosphatases, since the reaction has a $\Delta G \sim 0$, otherwise the reverse reaction displayed could also occur.

A

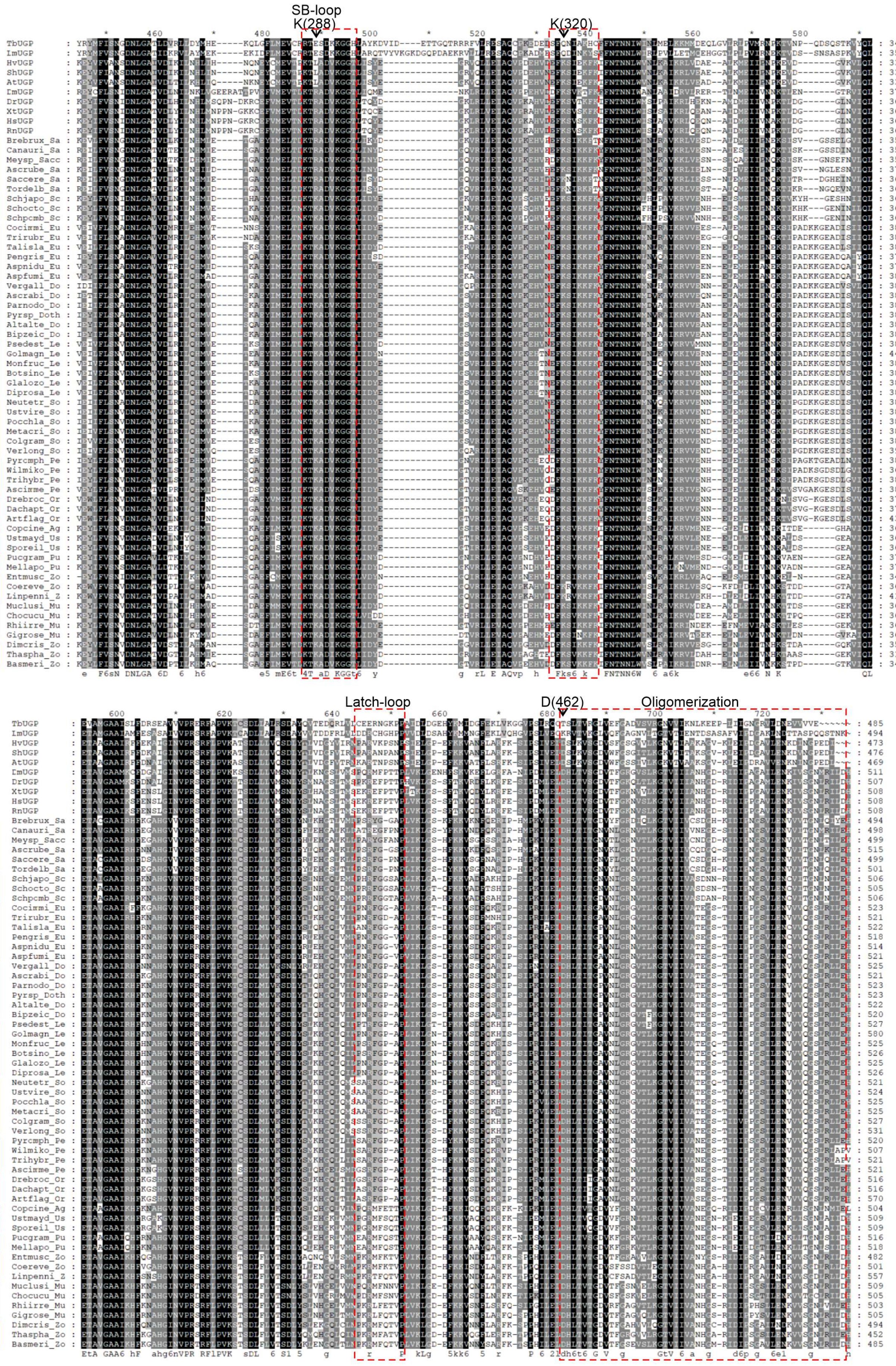

B

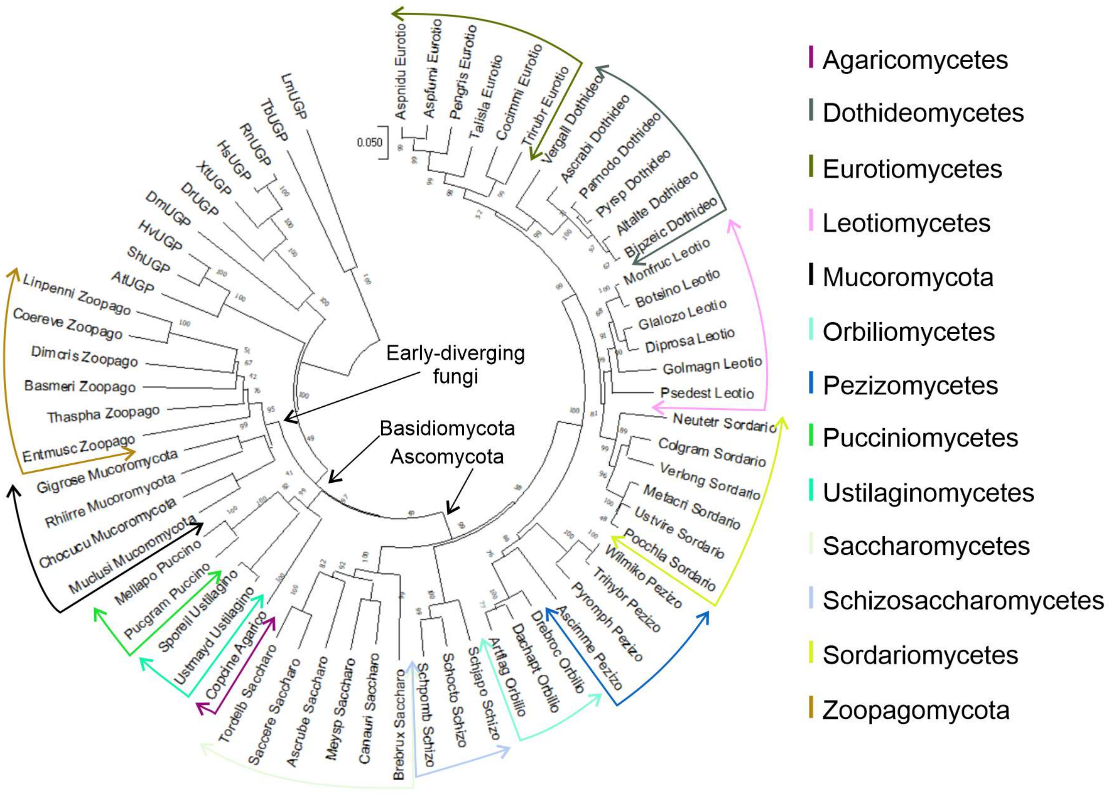

Supplemental Fig. S7. Evolutionary analyses of UGPs. (**A**) Alignment (Clustal omega) of the central and C-terminal domains of the UGP homologs listed in panel B. Specific domains/motifs and amino acids are highlighted in red. (**B**) Neighbor-joining tree (bootstrap of 5,000 replicates) for UGP homologs from species belonging to the classes of **Eurotiomycetes** (Aspnidu: *A. nidulans*, Aspfumi: *A. fumigatus*, Pengris: *Penicillium griseofulvum*, Talisla: *Talaromyces islandicus*, Cocimmi: *Coccidioides immitis*, Trirubr: *Trichophyton rubrum*), **Dothideomycetes** (Vergall: *Verruconis Gallopava*, Ascrabi: *Ascochyta rabiei*, Parnodo: *Phaeosphaeria nodorum*, Pyrsp: *Pyrenophora sp.*, Altalte: *Alternaria alternata*, Bipzeic: *Bipolaris zeicola*), **Leotiomycetes** (Monfruc: *Monilinia fructicola*, Botsino: *Botrytis sinoallii*, Glalozo: *Glarea lozoyensis*, Diprosa: *Diplocarpon rosae*; Golmagn: *Golovinomyces magnicellulatus*, Psedest: *Pseudogymnoascus destructans*), **Sordariomycetes** (Neutetr: *Neurospora tetrasperma*, Colgram: *Colletotrichum graminicola*, Verlong: *Verticilliuim longisporum*, Metacri: *Metarhizium acridum*, Ustvire: *Ustilaginoidea virens*, Pocchla: *Pochonia chlamydosporia*), **Pezizomycetes** (Wilmiko: *Wilcoxina mikolae*, Trihybr: Trichophaea hybrida, Pyromph: *Pyronema omphalodes*, Ascimme: *Ascobolus immersus*), **Orbiliomycetes** (Drebroc: *Drechslerella brochopaga*, Dachapt: *Dactylellina haptotyla*, Artflag: *Arthrobotrys flagrans*), **Schizosaccharomycetes** (Schjapo: *Schizosaccharomyces japonicus*, Schocto: *S. octosporus*, Schpomb: *S. pombe*), **Saccharomycetes** (Brebrux: *Brettanomyces bruxellensis*, Canauri: *Candida auris*, Meysp: *Meyerozyma sp.*, Ascrube: *Ascoidea rubescens*, Saccere: *Saccharomyces cerevisiae*, Tordelb: *Torulaspora delbrueckii*), **Agaricomycetes** (Copcine: *Coprinopsis cinerea*), **Ustilaginomycetes** (Ustmayd: *Ustilago maydis*, Sporeil: *Sporisorium reilianum*), **Pucciniomycetes** (Pucgram: *Puccinia graminis*, Mellapo: *Melampsora larici-populina*), and to **Mucoromycota** (Gigrose: *Gigaspora rosea*, Rhiirre: *Rhizophagus irregularis*, Chocucu: *Choanephora cucurbitarum*, Muclusi: *Mucor lusitanicus*) or **Zoopagomycota** (Entmusc: *Entomophthora muscae*, Thaspha: *Thamnocephalis sphaerospora*, Basmeri: *Basidiobolus meristosporus*, Dimcris: *Dimargaris cristalligena*, Coereve: *Coemansia reversa*, Linpenni: *Linderina pennispora*), compared to UGP homologs from *Leishmania major*, *Trypanosomas brucei*, *Homo sapiens*, *Xenopus tropicalis*, *Rattus norvegicus*, *Drosophila melanogaster*, *Danio rerio*, *Hordeum vulgare*, *Saccharum* hybrid cultivar, *Arabidopsis thaliana*.

Supporting References

1. Führing JI, Cramer JT, Schneider J, Baruch P, Gerardy-Schahn R, Fedorov R. 2015. A quaternary mechanism enables the complex biological functions of octameric human UDP-glucose pyrophosphorylase, a key enzyme in cell metabolism. Sci Rep 5:9618.
2. Führing J, Damerow S, Fedorov R, Schneider J, Münster-Kühnel A-K, Gerardy-Schahn R. 2013. Octamerization is essential for enzymatic function of human UDP-glucose pyrophosphorylase. Glycobiology 23:426–437.
3. Yu Q, Zheng X. 2012. The crystal structure of human UDP-glucose pyrophosphorylase reveals a latch effect that influences enzymatic activity. Biochem J 442:283–291.
4. Sanchez-Garcia R, Gomez-Blanco J, Cuervo A, Carazo JM, Sorzano COS, Vargas J. 2021. DeepEMhancer: a deep learning solution for cryo-EM volume post-processing. Commun Biol 4:874.
5. Cardone G, Heymann JB, Steven AC. 2013. One number does not fit all: Mapping local variations in resolution in cryo-EM reconstructions. J Struct Biol 184:226–236.
6. Roeben A, Plitzko JM, Körner R, Böttcher UMK, Siegers K, Hayer-Hartl M, Bracher A. 2006. Structural basis for subunit assembly in UDP-glucose Pyrophosphorylase from *Saccharomyces cerevisiae*. J Mol Biol 364:551–560.
7. Cotrim CA, Soares JSM, Kobe B, Menossi M. 2018. Crystal structure and insights into the oligomeric state of UDP-glucose pyrophosphorylase from sugarcane. PLoS One 13:e0193667.
8. McCoy JG, Bitto E, Bingman CA, Wesenberg GE, Bannen RM, Kondrashov DA, Phillips GN. 2007. Structure and dynamics of UDP–Glucose Pyrophosphorylase from *Arabidopsis thaliana* with bound UDP–Glucose and UTP. J Mol Biol 366:830–841.
9. Cramer JT, Führing JI, Baruch P, Brütting C, Knölker H-J, Gerardy-Schahn R, Fedorov R. 2018. Decoding allosteric networks in biocatalysts: Rational approach to therapies and biotechnologies. ACS Catal 8:2683–2692.
10. Mariño K, Güther MLS, Wernimont AK, Amani M, Hui R, Ferguson MAJ. 2010. Identification, subcellular localization, biochemical properties, and high-resolution crystal structure of *Trypanosoma brucei* UDP-glucose pyrophosphorylase. Glycobiology 20:1619–1630.
11. Raimi OG, Hurtado-Guerrero R, Borodkin V, Ferenbach A, Urbaniak MD, Ferguson MAJ, van Aalten DMF. 2020. A mechanism-inspired UDP-N-acetylglucosamine pyrophosphorylase inhibitor. RSC Chem Biol 1:13–25.
12. Edwards TE, Gardberg AS, Phan IQH, Zhang Y, Staker BL, Myler PJ, Lorimer DD. 2015. Structure of uridine diphosphate N-acetylglucosamine pyrophosphorylase from *Entamoeba histolytica*. Acta Crystallogr Sect F 71:560–565.
13. Peneff C, Ferrari P, Charrier V, Taburet Y, Monnier C, Zamboni V, Winter J, Harnois M, Fassy F, Bourne Y. 2001. Crystal structures of two human pyrophosphorylase isoforms in complexes with UDPGlc(Gal)NAc: role of the alternatively spliced insert in the enzyme oligomeric assembly and active site architecture. EMBO J 20:6191–6202.
14. Maruyama D, Nishitani Y, Nonaka T, Kita A, Fukami TA, Mio T, Yamada-Okabe H, Yamada-Okabe T, Miki K. 2007. Crystal structure of uridine-diphospho-N-acetylglucosamine pyrophosphorylase from *Candida albicans* and catalytic reaction mechanism. J Biol Chem 282:17221–17230.
15. Zhang Z, Bulloch EMM, Bunker RD, Baker EN, Squire CJ. 2009. Structure and function of GlmU from *Mycobacterium tuberculosis*. Acta Crystallogr Sect D 65:275–283.
16. Swift R V, Ong CD, Amaro RE. 2012. Magnesium-induced nucleophile activation in the guanylyltransferase mRNA capping enzyme. Biochemistry 51:10236–10243.
